# Network-based insights into miRNA regulation of β-cell insulin secretion in type 2 diabetes

**DOI:** 10.1016/j.isci.2025.114200

**Published:** 2025-11-22

**Authors:** Elaine Cowan, Alexandros Karagiannopoulos, Alessio Pollastri, Akira Asai, Mototsugu Nagao, Marlena Maziarz, Jonathan L.S. Esguerra, Lena Eliasson

**Affiliations:** 1 Unit of Islet Cell Exocytosis, Lund University Diabetes Centre (LUDC) Department of Clinical Sciences Malmö, Lund University, Lund, Sweden; 2 Bioinformatics Unit, Lund University Diabetes Centre (LUDC) Department of Clinical Sciences Malmö, Lund University, Lund, Sweden; 3 Clinical Research Centre, Skåne University Hospital, Malmö, Sweden; 4 Department of Endocrinology, Metabolism and Nephrology, Graduate School of Medicine, Nippon Medical School, Tokyo, Japan; 5 Science for Life Laboratory, Department Clinical Sciences Malmö, Lund University, Lund, Sweden

**Keywords:** health sciences, biological sciences, molecular mechanism of gene regulation, cell biology

## Abstract

Increasing evidence suggests that microRNAs (miRNAs) contribute to pancreatic β-cell compensation during type 2 diabetes (T2D) pathogenesis. To examine miRNA-mRNA interactions in human islets and their roles in β-cell insulin secretion and T2D, we performed small-RNA sequencing on pancreatic islets from nine individuals with T2D and 52 non-diabetic controls. We identified 70 differentially expressed miRNAs, with miRNAs upregulated in T2D enriched in a co-expression network associated with insulin secretion. Eight such upregulated miRNAs, including miR-101-3p and miR-9-5p associated with both first- and second-phase insulin secretion. Among them, miR-101-3p had the most mRNA targets, while highly abundant mRNA transcripts (e.g., *INS*) were regulated by few miRNAs. Overexpression of miR-101-3p in β-cells increased insulin release *in vitro* and reduced expression of CADM1, a target of miR-101-3p. In summary, we have comprehensively identified miRNA-mRNA alterations in human islets associated with T2D pathogenesis and propose that miR-101-3p plays an important role in β-cell insulin secretion.

## Highlights

• Seventy miRNAs are differentially expressed (DE) in islets from T2D vs*.* ND individuals
• These DE miRNAs are associated with phenotypes and gene targets linked to T2D
• Eight DE miRNAs are associated with both first- and second-phase insulin secretion
• MiR-101-3p regulates CADM1 expression, contributing to β-cell compensation

## Graphical abstract

**Figure:**
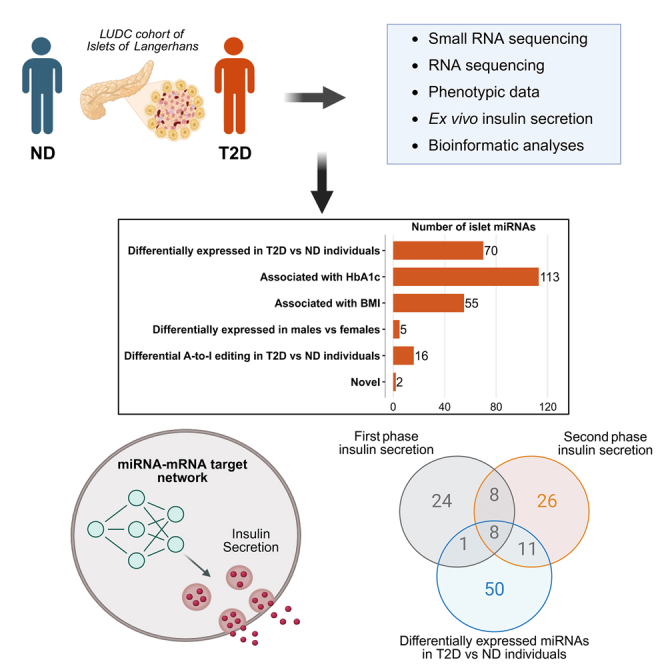

## Introduction

Lifestyle changes have led to an increasing number of people developing metabolic diseases, including type 2 diabetes (T2D), with an estimated 853 million people expected to have diabetes by 2050.^1^ The disease is characterized by impaired insulin secretion from pancreatic β-cells and peripheral insulin resistance. Its pathogenesis involves β-cell compensation to meet the increased insulin demand driven by insulin resistance. To develop more effective treatments, it is crucial to better understand the molecular mechanisms underlying β-cell insulin secretion and the reasons for its failure in T2D. We hypothesize that microRNAs (miRNAs) play a key role in this process.

Insulin secretion by β-cells occurs through Ca^2+^-dependent exocytosis, initiated by an influx of Ca^2+^ via voltage-sensitive Ca^2+^ channels. These channels open after the closure of ATP-dependent K^+^ (K_ATP_) channels and membrane depolarization. The K_ATP_ channels are sensitive to elevated ATP levels resulting from increased metabolic activity upon glucose uptake.^2^ A stepwise increase in glucose triggers a biphasic insulin response: a rapid first phase lasting 5–10 min, followed by a slower, pulsatile second phase. In T2D pathogenesis, first-phase insulin secretion is either repressed or absent, while second-phase insulin secretion is reduced.^3^ The inhibition of the first-phase insulin secretion has been linked to a reduced number of docked and primed insulin granules, though other contributing factors have also been proposed.^4^^,^^5^

MiRNAs are small non-coding RNAs that regulate post-transcriptional expression of specific target proteins by binding to sites primarily located in the 3′ untranslated region (3′UTR) of target messenger RNAs. MiRNAs have been suggested to act as rheostats, fine-tuning protein output.^6^ The first miRNAs identified in pancreatic islets and β-cells were found in rodents and included miR-375^7^ and miR-9.^8^ MiR-375 has been shown to impact Ca^2+^-dependent exocytosis and insulin biosynthesis,^7^^,^^9^ and its knockout in mice leads to reduced β-cell mass.^10^ MiR-9 was shown to inhibit the expression of the transcription factor Onecut-2, a positive regulator of insulin exocytosis; thus, miR-9 overexpression results in reduced insulin release.^8^ In addition, miR-9 has since been shown to be regulated by glucose and to target *SIRT1*, a NAD-dependent protein deacetylase important in the metabolic regulation of the β-cell.^11^

In the last 10 years, more than 30 islet miRNAs have been implicated in processes related to insulin secretion and T2D pathogenesis in humans.^12^ Most studies to date have investigated the impact of individual miRNAs (e.g., miR-200c,^13^ miR-125b,^14^ and miR-7^15^), highlighting their potential as therapeutic targets.^12^ However, these studies fall short in describing the full complexity of the miRNA-mRNA network, where one miRNA can regulate the expression of hundreds of target proteins, and a single protein may be regulated by multiple miRNAs. Identifying miRNAs that modulate insulin secretion by regulating multiple targets within the same signaling pathway (or several) may therefore offer a more effective pharmacological approach^12^ for prevention or treatment of T2D.

In this study, we aimed to elucidate the underlying complex network of the relationships between differential miRNA expression in T2D and glucose-stimulated insulin secretion. Using human pancreatic islets from 61 individuals (*n* = 9 T2D, *n* = 52 non-diabetic [ND] controls), we performed global small-RNA sequencing (sRNA-seq) and compared miRNA expression in those with T2D compared to controls. Additionally, we analyzed mRNA targets to identify islet-specific miRNA-mRNA pairs, investigated the association of those miRNA-mRNA pairs with first- and second-phase insulin secretion response and validated these findings in selective *in vitro* functional studies.

## Results

### Differentially expressed miRNAs in T2D and their association with insulin secretion

#### miRNA associations with first- and second-phase insulin secretion in human islets

**Figure 1:**
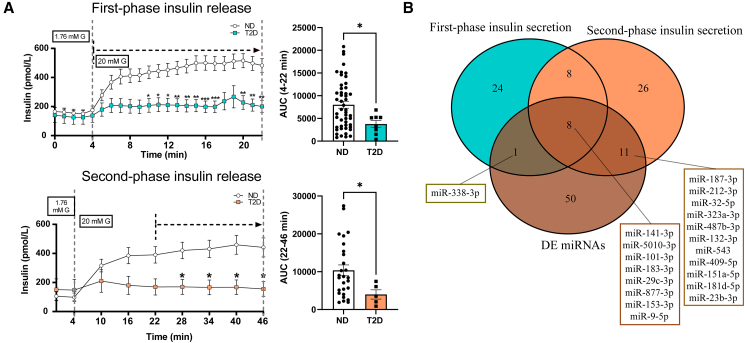
Association of miRNAs with first- and second-phase insulin secretion (A) First-phase (top images) and second-phase (bottom images) insulin release curves and a comparison of their corresponding areas under the curve (AUC) between T2D and ND donor islets. Islets were subjected to dynamic glucose perifusion at low (1.76 mmol/L) and high (20 mmol/L) glucose concentrations. First-phase insulin release was taken as the insulin release in the first 18 min after the initial perifusion with high glucose solution (minutes 4–22) and was measured in *n* = 49 ND/*n* = 8 T2D donors. The second-phase insulin release was taken from the 24 min period starting at minute 22 after initial perifusion with high glucose solution (minutes 22–46) and was measured in *n* = 27 ND/*n* = 5 T2D donors. Plots represent islet perifusion data, the points and error bars indicate the mean ± SEM of insulin concentration (in pmol/L) at a given time point. The data were analyzed via two-way ANOVA with Bonferroni’s post hoc test (insulin curves) and Mann-Whitney test (AUC graphs). ∗*p* < 0.05, ∗∗*p* < 0.01, ∗∗∗*p* < 0.001. (B) Venn diagram summarizing the number of miRNAs found to be associated with first- and second-phase insulin release AUCs. Of these, 20 were found to be differentially expressed (DE) in T2D compared to ND and are displayed in the figure.

To identify miRNAs associated with insulin secretion, we first evaluated the first- and second-phase insulin secretion curves after subjecting islets from ND and T2D individuals (Table S1) to dynamic glucose perifusion. First- and second-phase insulin secretion was significantly reduced in islets from T2D donors compared to those from ND donors. The insulin secretion over time in the first and second phase, as well as the areas under the curves (AUC), is shown in Figure 1A. We then estimated the association between miRNA expression in the islets and insulin secretion AUC separately for both first- and second-phase insulin secretion. We found 41 miRNAs to be associated (at *p* < 0.05) with first-phase insulin secretion and 53 with second-phase insulin secretion (Table S2), of which 16 were common to both phases.

#### Differentially expressed miRNAs in T2D and their association with insulin secretion

Eight differentially expressed (DE) miRNAs in islets of T2D individuals compared to ND controls (from now on referred to as DE miRNAs) were associated with both first- and second-phase insulin secretion (Figure 1B). One miRNA, miR-338-3p, was DE in T2D and found to be associated only with first-phase insulin secretion, and 11 DE miRNAs were associated only with the second phase (Figures 1B; Table S2).

**Figure 2:**
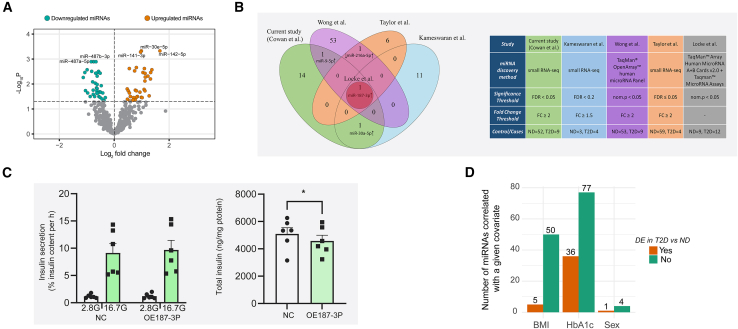
Human islet miRNA expression in T2D and correlation with donor covariates (A) Volcano plot showing the log2-transformed fold change (*x* axis) vs. the -log10-transformed *p*_FDR_ (*y* axis) of miRNA expression in islets from T2D compared to ND donors. The horizontal dashed line indicates the -log_10_-transformed statistical significance threshold of *p*_FDR_ = 0.05 and the miRNAs we consider to be DE are shown in green (if downregulated) and orange (if upregulated). The vertical dashed line corresponds to the log_2_ fold change in expression equal to 0 (i.e., no change) that separates up- and downregulated miRNAs. (B) Comparison of study designs of our (current) and four previous studies that investigated differences in the miRNA expression in T2D vs. ND human islets (right image). A venn diagram summarizing overlaps and discrepancies between the DE miRNAs identified in our study and the four others (left image). (C) Glucose-stimulated insulin secretion (left image) and insulin content (right image) in negative control (NC) cells and cells overexpressing miR-187-3p (OE187-3p) INS-1 832/13. Insulin secretion was measured at two glucose concentrations: 2.8G (2.8 mmol/L glucose) and 16.7G (16.7 mmol/L glucose). Data are presented as mean ± SEM. Statistical analysis was performed using two-way ANOVA with Šídák’s multiple comparisons test (left image) and paired Student’s t test (right image), ∗*p* < 0.05. Statistically significant differences were observed between 2.8G versus 16.7G stimulation but were not shown in the figure for clarity. (D) The number of DE and non-DE miRNAs in T2D compared to ND islets, with expression levels associated with sex, BMI, and HbA1c levels of the donors. Associations are said to be statistically significant if a *p* value based on a likelihood ratio test and a linear regression model with robust standard errors is <0.05.

To study the association between the DE miRNAs and glucose-stimulated insulin secretion, we first performed differential expression analysis comparing the expression of miRNAs in pancreatic islets from 9 T2D individuals and 52 ND controls. MiRNA expression was quantified using sRNA-seq in human pancreatic islets and sequencing yielded high-quality data, with approximately 70% of the mapped reads assigned to mature miRNA sequences (Figure S1). We identified 70 DE miRNAs (with a false discovery rate [FDR] controlled at 5% [*p*_FDR_ < 0.05]), and of these, 34 were upregulated and 36 downregulated in islets from T2D donors compared to ND donors (Figure 2A; Table S3). Of all DE miRNAs, three had differential A-I modification patterns (miR-1247-5p, miR-494-3p, and miR-497-5p; Figure S2). A-to-I modifications are the most common type of post-transcriptional RNA editing in humans in which adenosine is converted to inosine (A to I).

#### Enrichment of 34 upregulated T2D-associated miRNAs in miRNA-disease databases

To validate our findings, we performed a miRNA-disease enrichment analysis of the 70 miRNAs we identified to be DE in our T2D samples and found that 20 are known to be associated with T2D in miRNA-disease association databases (Table S4). To perform this analysis, we used data from two such databases, the RNADisease v4.0^16^ and the Human MicroRNA Disease Database (HMDD v3.0^17^), and in both databases, the term “type 2 diabetes” was significantly enriched for only the 34 upregulated, but not the 36 downregulated DE miRNAs after a hypergeometric test (RNADisease *p*_FDR_ = 8.9 × 10^−8^, HMDD *p*_FDR_ = 5.8 × 10^−5^; Table S4). Some (*n* = 9) of these upregulated miRNAs have only been associated with T2D as circulatory T2D biomarkers, though their functions in the islets have not been described.^16^^,^^17^

#### MiR-187-3p is identified as a consistently upregulated miRNA in T2D in a cross-study comparison

Comparing our set of DE miRNAs with the DE miRNAs characterized in four previous studies investigating DE miRNAs in T2D,^18^^,^^19^^,^^20^^,^^21^ we found one miRNA (miR-187-3p) to be reported as upregulated in T2D compared to ND in all five studies (Figure 2B). An *in vitro* analysis of miR-187 overexpression in INS-1 832/13 cells (Figure S3A) did not show an effect on glucose-stimulated insulin secretion (Figure 2C) but showed reduced insulin content (Figure 2C). Additionally, two miRNAs, miR-9-5p and miR-30a-5p, were upregulated in both our study and one other study. However, most of the identified miRNAs in all five studies remain unconfirmed (Figure 2B). The study designs of our and the four previous studies are summarized in Figure 2B.

### Associations of miRNA expression with HbA1c, BMI, and sex

We next analyzed the association between islet miRNA expression with HbA1c, body mass index (BMI), and sex of the human donors (Figures 2D; Table S5). Expression of 113 miRNAs was associated with HbA1c levels, of these, 36 (30%) were DE in T2D compared to ND, indicating a possible role of these miRNAs in T2D pathogenesis. The expression of 55 miRNAs was associated with BMI, of which 5 (∼10%) were DE in T2D compared to ND. Furthermore, the expression of five miRNAs differed between males and females (*n* = 38 male [33 ND/5 T2D] and 23 female [19 ND/4 T2D]) donors, with four belonging to the miR-181 family. Among these, miR-181d-5p was DE in T2D compared to ND.

### Regulatory networks in T2D are enriched for mRNA targets of DE miRNAs

#### Identification of mRNA targets of DE miRNAs in T2D suggests differential targeting patterns

**Figure 3:**
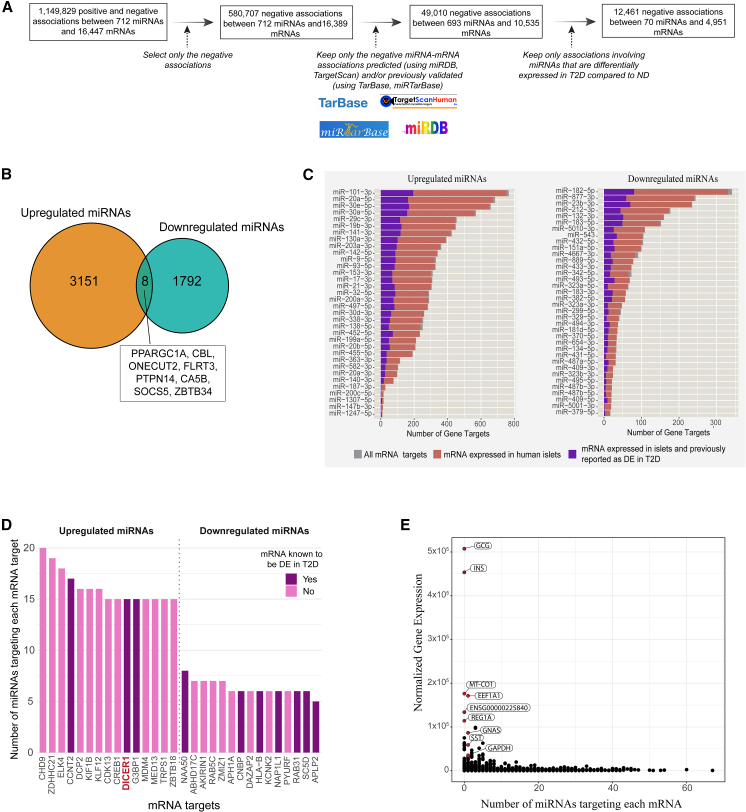
Association analysis between miRNAs and their potential mRNA targets in human islets (A) Schematic of the miRNA-mRNA target identification workflow. Briefly, associations between the expression of miRNAs and mRNAs were defined with a likelihood ratio test (nominal *p* value < 0.05), and negative associations were scanned across publicly available miRNA-mRNA target data to discover validated and/or predicted associations. Lastly, only the miRNA-mRNA pairs consisting of DE miRNAs in T2D were used for downstream analysis. (B) Venn diagram demonstrating common mRNA targets of upregulated and downregulated miRNAs in T2D. (C) Bar charts representing the number of mRNA targets associated with the differentially upregulated (left) and downregulated (right) miRNAs in T2D, and the proportion of mRNA targets that are expressed in human islets and have been previously reported as DE in T2D. (D) mRNA targets (previously repoted as DE in T2D or not) targeted by the highest number of upregulated (up) and downregulated (down) miRNAs in T2D. (E) Dot plot representing the human islet expression level of mRNAs (normalized Deseq2 counts) in relation to the number of miRNAs that target them. Selected mRNAs that are highly expressed and targeted by no or few miRNAs are displayed.

The approach we used to identify potential mRNA targets of the 70 DE miRNAs in human islets is described in Figure 3A. In total, we identified 4,951 mRNA targets which were negatively associated with these 70 DE miRNAs, and whose interactions were either predicted or validated by external databases (Table S6). The 34 miRNAs upregulated in T2D were associated with almost double the number of mRNA targets (3,159 mRNAs) compared to the 36 miRNAs downregulated in T2D (1,800 mRNAs). Eight mRNAs were commonly targeted by both upregulated and downregulated DE miRNAs (Figure 3B). The miRNA associated with the largest number of targets was miR-101-3p (associated with 766 mRNAs; Figure 3C).

#### T2D-related genes are enriched among mRNA targets of DE miRNAs in human islets

To investigate which of the 4,951 mRNA targets were implicated in T2D, we compiled a comprehensive list of 3,216 genes known to be associated with T2D from eight bulk- and single-cell studies that compared islet transcriptomes between T2D and ND donors^22^^,^^23^^,^^24^^,^^25^^,^^26^^,^^27^^,^^28^^,^^29^ (Table S7A). The 34 upregulated DE miRNAs were associated with a larger number of T2D-related genes (*n* = 663) than the 36 downregulated DE miRNAs (*n* = 461) (Figure 3C). Approximately 25% of the mRNAs targeted by the DE miRNAs were previously reported to be DE in T2D compared to islets from ND individuals (Figure 3C). To identify enriched targeting of miRNAs to mRNAs previously reported as DE in T2D, we performed an enrichment analysis including two sets: the “universe” set consisting of the 10,535 mRNAs and the 693 miRNAs from the 49,010 negatively associated miRNA-mRNA pairs (Figure 3A, step 3) and the 3,216 genes previously reported as related to T2D (Table S7A). Using a hypergeometric test, we identified enriched targeting of 1,617 mRNAs by 167 miRNAs (at *p*_FDR_ < 0.05), and 41 of these were among our 70 DE miRNAs (Table S7B).

#### DE miRNAs target key regulatory genes including DICER1, but rarely target highly expressed islet genes

We identified 4,951 mRNAs as targeted by the 70 DE miRNAs (Figure 3A, last step), with 2,570 of these being targets of more than one miRNA. For example, the regulator of miRNA biogenesis, *DICER1,* is targeted by at least 15 upregulated miRNAs (Figure 3D). The large number of miRNAs targeting *DICER1* might indicate that the regulation of *DICER1* expression is tightly controlled by miRNAs through regulatory feedback loops.^30^^,^^31^ This would be an interesting topic for future studies. We also found that the mRNAs are targeted by a lower number of downregulated DE miRNAs in T2D compared to upregulated DE miRNAs (Figure 3D). Additionally, very highly expressed genes in the human islet (e.g., *INS* and *GCG* coding for insulin and glucagon, respectively) are targeted by few or zero miRNAs (Figure 3E).

### Co-expression clustering reveals enrichment of insulin secretion pathways among mRNA targets of upregulated miRNAs

**Figure 4:**
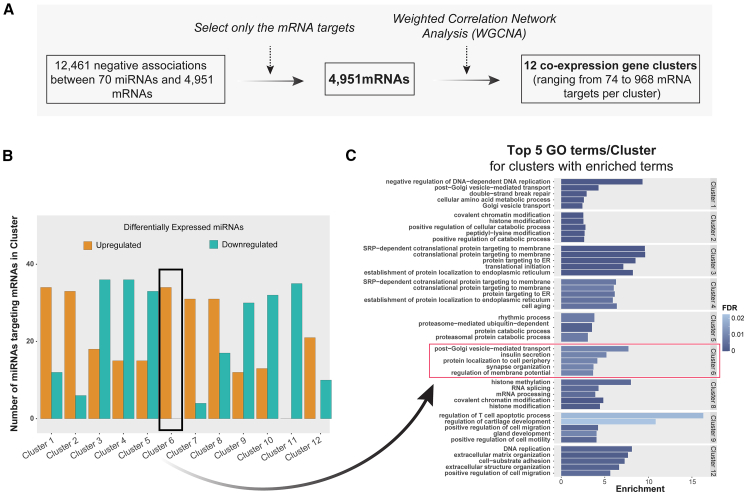
Co-expression networks of miRNA mRNA targets in human islets (A) Schematic of the weighted correlation network analysis (WGCNA) in mRNA targets of miRNAs DE in T2D. (B) Proportion of upregulated and downregulated miRNAs in T2D associated with the co-expressed mRNAs included in each co-expression cluster. (C) Pathway enrichment analysis revealed the top 5 GO-enriched terms of the cluster genes ranked by increasing *p*_FDR_. Only clusters with enriched pathway terms (enrichment threshold *p*_FDR_ < 0.05) are shown.

Next, the 4,951 mRNA targets associated with the 70 DE miRNAs (Figure 3A, last step) were grouped into 12 clusters based on co-expression using weighted correlation network analysis (WGCNA)^32^ (Figure 4A; Table S8). The proportion of upregulated and downregulated miRNAs associated with the mRNA targets in each cluster varied significantly. Cluster 6 was exclusively linked to upregulated miRNAs, and cluster 11 to downregulated miRNAs (Figure 4B). Each cluster was then functionally annotated using gene ontology (GO) and analyzed for enrichment of molecular pathways (at *p*_FDR_ < 0.05). Among the top five GO terms enriched in cluster 6 (by lowest *p*_FDR_) were: “insulin secretion,” “synapse organization,” and “regulation of membrane potential”—pathways not enriched in any other cluster (Figures 4C; Table S9). That cluster consisted of 238 mRNAs, 67 of which previously reported as T2D-related. The observation that only upregulated miRNAs in T2D were associated with mRNAs representing genes involved in insulin secretion aligns with our previous finding suggesting upregulated miRNAs play a more prominent role in T2D characterization compared to downregulated miRNAs.^33^

### Functional validation of miR-9-5p and miR-101-3p in insulin-secreting cell lines

#### Impact of miR-9-5p and miR-101-3p overexpression on insulin secretion and content

To validate our bioinformatics findings *in vitro*, we focused on two of the most promising miRNA candidates, miR-9-5p and miR-101-3p. MiR-9-5p was DE in T2D (Table S3) and was the only miRNA associated with both phases of insulin secretion (first phase: *p*_LRT___FDR_ = 0.0006 and second phase: *p*_LRT___FDR_ = 0.005; Table S2). MiR-101-3p was DE in T2D (Table S3) and was associated with second phase (*p*_LRT___FDR_ = 0.037) and had a tendency toward an association with first phase (*p* = 0.004, *p*_LRT___FDR_ = 0.12) (Table S2).

**Figure 5:**
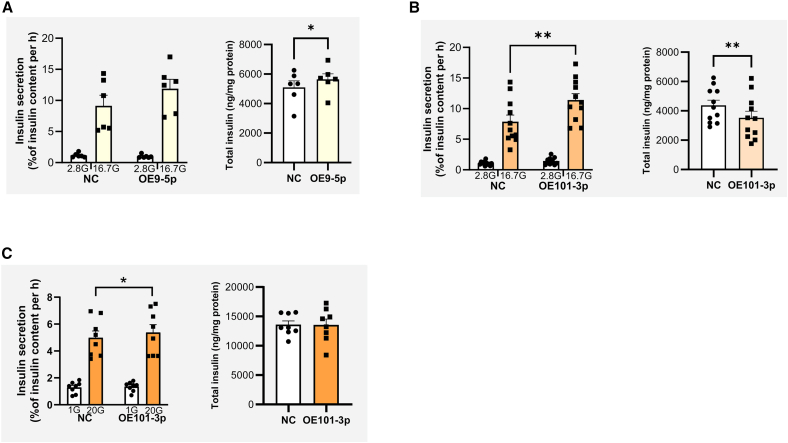
Glucose-stimulated insulin secreion after overexpression of miR-9-5p and miR-101-3p (A) Glucose-stimulated insulin secretion (left) and insulin content (right) in NC and OE9-5p INS-1 832/13 cells. (B) As in (A), but OE101-3p cells. (C) As in (B), but in EndoC-βH1 cells. All data are presented as mean ± SEM. Statistical analysis for the insulin secretion measurements in INS-1 832/13 cells was performed using two-way ANOVA with Šídák’s multiple comparisons test and for all other measurements paired Student’s t test. ∗*p* < 0.05; ∗∗*p* < 0.01; Significant differences occurred between 2.8G/1G and 16.7G/20G stimulation in (A, B, C), but were not added in the figure for clarity. 2.8G – 2.8 mmol/L glucose; 16.7G –16.7 mmol/L glucose; 1G – 1 mmol/L glucose; 20G – 20 mmol/L glucose; NC – negative control; OE9-5p – overexpression of miR-9-5p; OE101-3p – overexpression of miR-101-3p.

We performed selective functional experiments using INS-1 832/13 cells in which miR-9-5p or miR-101-3p was overexpressed (OE9-5p and OE101-3p cells). After verifying an increased expression of each miRNA by qPCR (Figures S3B and S3C), we performed a glucose-stimulated insulin secretion assay. Glucose-stimulated insulin secretion in OE9-5p cells did not differ from that in negative control cells, but intracellular insulin content was elevated (Figure 5A). In OE101-3p cells, insulin secretion was increased while the insulin content was reduced (Figure 5B). Given that altered glucose-stimulated insulin secretion was only observed in OE101-3p cells and that miR-101-3p was not previously well-studied in the context of human islets, we repeated this experiment using the human cell line EndoC-βH1 (Figures 5C, S3D). Consistent with the INS-1 832/13 cell data, overexpression of miR-101-3p in EndoC-βH1 cells also led to increased insulin secretion (Figure 5C).

### Interaction analysis of miR-9-5p and miR-101-3p and their mRNA targets

**Figure 6:**
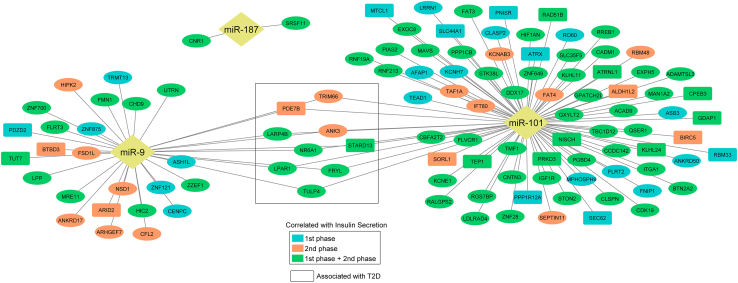
miRNA-mRNA target network for miR-9-5p and miR-101-3p The network consists of mRNA targets significantly associated with first-phase and/or second-phase insulin secretion AUC values. mRNAs inside rounded rectangular boxes were previously annotated as DE in T2D. Common mRNA targets between miR-9 and miR-101 are indicated by a rectangle in the center of the plot.

We then visualized the miRNA-mRNA interactions (at *p* < 0.05) for miR-9-5p and miR-101-3p. As each of these miRNAs has numerous targets, we decided to retain only the ones whose expression was associated with first- and second-phase insulin secretion (Table S10). Overall, a total of 108 mRNA targets were identified: nine were common to both miRNAs, 75 targets of miR-101-3p, and 24 targets of miR-9-5p were associated with first- and second-phase insulin secretion in our analysis. Of the 108, 24 are known to be associated with T2D (Figure 6).

Out of the 108 unique mRNA targets, 106 were reported to be expressed in human β-cells in a published human islet single-cell transcriptome dataset,^34^ supporting their involvement in β-cell processes (Tables S10 and S3 [Beta_Cell_expression]). For miR-101-3p, 64% (54/84) of mRNA targets were associated with both phases of insulin secretion, that same proportion for miR-9-5p was 52% (17/33).

### MiR-101-3p regulates Cadm1 expression under glucotoxic and glucolipotoxic conditions

**Figure 7:**
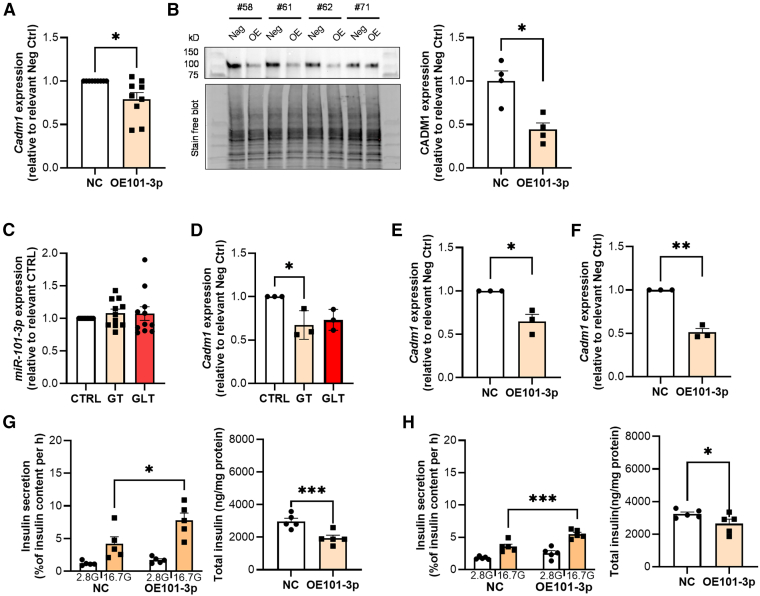
Impact of overexpression of selected microRNAs on glucose-stimulated insulin secretion (A) Gene expression of *Cadm1* in NC and OE101-3p INS-1 832/13 cells. (B) Western blot of CADM1 in four passages of INS-1 832/13 cells after transfection with NC (Neg) and OE101-3p (OE). Summary of all four biological replicates to the right. (C) miR-101-3p expression in GT and GLT. (D) *Cadm1* expression in GT and GLT. (E and F) Cadm1 expression in NC and OE101-3p cells in GT (E) and GLT (F) as indicated. (G) Glucose-stimulated insulin secretion (left) and insulin content (right) in NC and OE101-3p INS-1 832/13 cells in GT. in GT. (H) As in (F) but in GLT. All data are presented as mean ± SEM. Statistical analysis for the insulin secretion measurements was performed using two-way ANOVA with Šídák’s multiple comparisons test and for all other measurements paired Student’s t test. ∗*p* < 0.05; ∗∗*p* < 0.01; ∗∗∗*p* < 0.001; Significant differences occurred between 2.8G and 16.7G in (F, G) but was not added in the figure for clarity. 2.8G – 2.8 mmol/L glucose; 16.7G – 16.7 mmol/L glucose; NC – negative control; OE101-3p – overexpression of miR-101-3p; CTRL-control 5.5 mM glucose; GT – glucotoxic condition (16.7 mmol/L glucose 24h); GLT – glucolipotoxic condition (16.7 mmol/L glucose and 0.5 mmol/L palmitate 24 h).

Of the miR-101-3p targets associated with first- and second phase (Table S10), we decided to perform functional validation of eight, two significantly correlated with first phase and six with both phases, all enriched in β-cells. Only expression of *Cadm1* was reduced in OE101-3p INS-1 832/13 cells (Figure 7A,S4, and S5), where the miRNA targets a highly conserved site between humans and rats (Figure S6). Moreover, we verified reduced protein level of CADM1 in OE101-3p INS-1 832/13 cells (Figure 7B).

We next investigated whether the expression of miR-101-3p and *Cadm1* is affected by glucotoxic (GT) and glucolipotoxic (GLT) conditions in INS-1 832/13 cells (Figures 7C and 7D), since these conditions among others are associated with T2D and can reduce *Cadm1* expression.^35^ While miR-101-3p expression level did not appear to change after 24 h incubation under GT or GLT conditions (Figures 7C and S5), we could confirm that *Cadm1* expression was reduced in INS-1 832/13 (Figures 7D and S5), suggesting that factors other than miR-101-3p also influence the expression of *Cadm1*. Interestingly, expression of *Cadm1* was further reduced in OE101-3p cells incubated under GT and GLT conditions (Figures 7E, 7F, and S3E). Moreover, the reduced insulin secretion by the GT and GLT condition was partly counteracted by overexpression of miR-101-3p (Figures 7G and 7H).

### Abundant and putative miRNAs

The abundance analyses were consistent with previous miRNA microarray-based^33^^,^^36^ and sequence-based^18^^,^^20^ studies. Regardless of diabetic status, we found miR-375-3p, let-7b-5p, let-7a-5p, miR-125a-5p, and miR-7-5p to be the top five most abundant miRNAs in human islets (Figure S7A). Among the 15 most abundant miRNAs, 13 were common to both ND and T2D samples. The remaining two most abundant miRNAs for ND were miR-30d-5p and miR-103a-3p, while miR-141-3p and miR-200a-3p were highly expressed in T2D (Figure S7A; Table S11).

Lastly, we discovered the sequence and structure of 2 putative miRNAs (Figure S7B). Both miRNAs had a very high probability score (0.99/0.97) and were identified independently in most of the samples (58 and 52 out of 61 samples, respectively). Nevertheless, their expression levels were relatively low (mean normalized count = 65 and 91, respectively) and they were not DE in islets from ND and T2D donors.

## Discussion

This comprehensive investigation of DE miRNAs, their associations with insulin secretion, and the identification of their mRNA targets reveals how miRNA-mediated modulation of gene regulatory networks influences β-cell insulin secretion (Figure S8). We identified 70 DE miRNAs in human pancreatic islets from individuals with T2D compared to ND (34 upregulated and 36 downregulated in T2D). Many of these miRNAs were significantly associated with first- and second-phase glucose-stimulated insulin secretion, with eight DE miRNAs, including miR-9-5p and miR-101-3p, linked to both phases. Notably, miR-101-3p, which was upregulated in islets from T2D donors, had the highest number of mRNA targets among the 70 DE miRNAs. In our *in vitro* studies, overexpression of miR-101-3p in β-cells enhanced glucose-stimulated insulin secretion, suggesting that its upregulation in T2D may be involved in a compensatory mechanism to enhance insulin secretion. Upregulated DE miRNAs were enriched for known T2D-associated molecular functions and targeted a greater number of mRNAs, including key regulatory targets such as *DICER1* and *CADM1*. Moreover, functional characterization of clusters of co-expressed mRNA targets of upregulated miRNAs in T2D showed enrichment in pathways related to insulin secretion.

While sRNA-seq analysis identified 70 DE miRNAs, disease-enrichment analysis found T2D to be significantly enriched only in the 34 upregulated miRNAs. Similarly, miRNA-mRNA interaction analysis found that almost twice as many target mRNAs were predicted to interact with the upregulated miRNAs compared to the downregulated ones. Additionally, co-expression clustering of mRNA targets of DE miRNAs found that only upregulated miRNAs were linked to a cluster of mRNA targets associated with molecular pathways involved in insulin secretion. Among these were miRNAs such as miR-9, miR-29, miR-30d, miR-141, miR-130a, miR-200c, and miR-497, which have previously been shown to be regulators of insulin secretion.^13^^,^^37^^,^^38^ Moreover, miRNA targets that were part of this cluster and are known to be associated with insulin secretion include *RFX3*,^39^ *CLOCK*,^40^ *CACNA1D*,^41^ and *KCNB1*.^42^ Both compensatory increase and dysfunctional decrease of glucose-stimulated insulin secretion are likely scenarios for how miRNAs regulate β-cell function in human T2D pathogenesis.^12^ Our findings suggest that the altered functional output of β-cells is primarily mediated by upregulation, rather than downregulation, of specific miRNAs.

Among the upregulated miRNAs in human islets from T2D donors we identified miR-101-3p, which has not been previously described in human islets. This miRNA had the highest number of mRNA targets and was associated with both first- and second-phase insulin secretion. One of its targets, the growth suppressor CADM1, showed reduced expression at the protein level following overexpression of miR-101. *Cadm1* knockdown promotes β-cells proliferation^43^ and has been shown to increase glucose-stimulated insulin secretion through remodeling of the actin cytoskeleton and interaction with exocytotic proteins.^35^ Prior studies have shown reduced expression of *Cadm1* in ob/ob mice and regulation of *Cadm1* by Ago2 and miR-375.^10^^,^^43^ In our hands, *Cadm1* expression was reduced by glucose toxicity (GT; independent of miR-101), in line with previous data,^35^ whereas expression of miR-101-3p was not regulated by GT or GLT. Others have suggested a regulation of miR-101-3p expression by increased inflammation^44^ and, interestingly, overexpression of miR-101-3p resulted in further reduced expression of *Cadm1* in GT and GLT. Indeed, since expression of miR-101-3p was, as noted, not regulated by GT or GLT, and its overexpression enhanced glucose-stimulated insulin secretion under all tested conditions (control, GT, and GLT), miR-101-3p appears to have a compensatory function, at least partly mediated via CADM1.

We validated the recent finding by Wong et al. (2021) that miR-9-5p is DE in T2D.^21^ Moreover, our study showed miR-9-5p had the strongest correlation with both phases of insulin secretion. Our functional *in vitro* data provides further support for a role of miR-9-5p in the regulation of β-cell compensation, which contrasts with previous reports that miR-9 is a negative regulator of insulin secretion in rodent models.^8^^,^^45^ Notably, we found several mRNA targets commonly regulated by both miR-101-3p and miR-9-5p, including *PDE7B* (a modulator of glucose-stimulated insulin secretion^22^^,^^46^), *STARD13* (a component of the cytoskeletal organization in islets crucial for glucose-stimulated insulin secretion^47^), *ANK3* (known to regulate glucose-stimulated insulin secretion by directly interacting with several voltage-dependent channels^48^), and *SYT13* (a vesicle trafficking protein important for insulin secretion with reduced expression in islets of T2D individuals^49^^,^^50^).

Regarding first-phase insulin secretion specifically, we made two particularly interesting observations which connect the involvement of miRNAs to the final step of the exocytotic process of insulin granule fusion in β-cells.^4^ First, only one DE miRNA, miR-338-3p, was correlated exclusively with first-phase insulin secretion. Overexpression of miR-338-3p has been shown to downregulate PDX1 and reduce insulin secretion.^51^ PDX1 expression is positively correlated with exocytotic gene expression, which is downregulated in T2D human islets.^50^^,^^52^ Second, miR-335-5p, the miRNA with the most A-I modification sites in our data, is involved in the regulation of the final steps of the exocytotic process: the priming of insulin granules.^53^

There is substantial heterogeneity in DE miRNAs reported by previous large-scale studies. This is likely due to differences in islet handling protocols, identification methods/technologies used, sample size, and data analysis methods. Of the DE miRNAs identified in our study, miR-187-3p was the only one consistently reported across all earlier studies. However, our findings are supported by previous studies showing that several of the DE miRNAs identified here have been linked to T2D or β-cell function. For example, miR-183-3p, miR-183-5p, and miR-299-5p^21^^,^^54^ are among the downregulated miRNAs, and their knockdown has been shown to affect proinsulin transcription and β-cell viability. Additionally, several of the upregulated miRNAs in our dataset—including miR-9, miR-29, miR-30d, miR-141, miR-130a, and miR-200c^8^^,^^11^^,^^12^^,^^13^^,^^38^^,^^55^^,^^56^^,^^57^—have previously been implicated in the regulation of insulin secretion. These observations reinforce the biological relevance of our results.

Overexpression of miR-187-3p has been shown to reduce insulin secretion in rat islets,^19^ and we observed reduced insulin content in INS-1 832/13 cells. Moreover, we found miR-187-3p expression to be correlated with second-phase insulin secretion in human islets. In all, these findings suggest that the upregulation of miR-187-3p is part of a mechanism leading to dysfunctional glucose-stimulated insulin secretion in T2D.

In conclusion, we have demonstrated the potential involvement of several miRNAs in the pathogenesis of T2D. Many of these miRNAs, and their predicted mRNA targets, were significantly associated with impaired glucose-stimulated insulin secretion—a hallmark of β-cell dysfunction during development of T2D. Notably, miR-101-3p emerged as a strong candidate potentially involved in regulating the compensatory response to β-cell stress during T2D pathogenesis. These findings reveal a complex network of miRNA-mediated gene regulation contributing to β-cell dysfunction and compensation in T2D. Finally, the involvement of specific miRNAs such as miR-101-3p in these processes warrants further research to evaluate their potential as therapeutic targets for preserving or enhancing β-cell function in diabetes.

### Limitations of the study

One of the limitations of our study is that our sRNAseq was performed in whole islets comprising multiple cell types rather than single β-cells. Access to human islets is restricted to donors who are brain-dead, limiting tissue availability, and single-cell sRNA-sequencing remains challenging. Hence, we chose the most optimal approach of performing screening in islets and functional follow-up in β-cells. Our study stands out as the first to investigate the associations of islet miRNA expression changes with phasic insulin secretion *in vivo*. While the use of primary human β-cells would have been preferable for a functional follow-up, limited availability of human islets presents a significant challenge. Therefore, we used alternative cellular models in the study, specifically the EndoC.βH1 and INS-1 832/13 cell lines, which are widely used surrogates in β-cell research. The limited availability of human islets imposed additional constraints on this study, such as the absence of qPCR-based validation of the RNA sequencing results. Furthermore, since it was necessary to prioritize certain experiments, insulin content could not be measured in all donor islets. Consequently, we could not determine whether the miRNA-associated changes in insulin secretion observed in this cohort reflect reduced insulin content and/or impaired insulin secretion. In this regard, our conclusions rely solely on the results from our cell line experiments. Of note, another weakness is that the publicly available miRNA-mRNA target data used in our analyses were derived from various tissues and cell lines. While this was the case, we characterized both the miRNA and mRNA expression profiles from islets of the same individuals. This made it possible to reliably select database-derived miRNA-mRNA pairs relevant to our investigation in human islets.

## Resource availability

### Lead contact

Further information and requests for resources should be directed to and will be fulfilled by the lead contact, Lena Eliasson (lena.eliasson@med.lu.se).

### Materials availability

This study did not generate new unique materials.

### Data and code availability

• Raw and processed small RNA sequencing data are deposited in the Lund University Diabetes Center (LUDC) repository (https://www.ludc.lu.se/resources/repository) under the following name and accession number LUDC microRNA cohort (LUDC2025.08.2) and is available from the lead contact (Lena Eliasson) upon request. Data must be granted by the LUDC Human Tissue Laboratory steering committee.
• This paper does not report original code.
• Any additional information required to re-analyze the data reported in this paper is available from the lead contact upon request.

## Acknowledgments

We acknowledge Anna-Maria Ramsay, Malin Svensson, and Fong To for technical assistance. We thank the Nordic Network for Clinical Islet Transplantation and Human Tissue Laboratory within Exodiab/LUDC. The study was supported by grants from the Swedish Foundation for Strategic Research (IRC15-0067); the Swedish Research Council 2009-1039 (Exodiab); the Swedish Research Council 2019-01406 and 2024-03597 (L.E. project grants); the Region Skåne-ALF (L.E.); the Swedish Diabetes Foundation DIA2019-454 and DIA2022-723 (L.E.); the Japan Society for the Promotion of Science 22KK0281 (M.N.); and the Royal Physiographic society in Lund (A.K.). L.E. is a SciLife Lab group leader.

## Author contributions

E.C., conceptualization, data curation, formal analyses, investigation, methodology, visualization, writing – original draft, writing – review and editing; A.K., conceptualization, data curation, formal analyses, investigation, methodology, visualization, writing – original draft, writing – review and editing. A.P., investigation, writing – review and editing. A.A., investigation, writing – review and editing; M.N., investigation, writing – review and editing; M.M., data curation, formal analyses, methodology, writing – review and editing; J.L.S.E., conceptualization, writing – review and editing; L.E., conceptualization, funding acquisition, project administration, supervision, visualization, writing – original draft, writing – review and editing. E.C. and A.K. are co-first authors and have contributed equally to the work and appear in alphabetical order.

## Declaration of interests

The authors have declared that no conflict of interest exists.

## STAR★Methods

### Key resources table

**Table:** 

REAGENT or RESOURCE	SOURCE	IDENTIFIER
**Antibodies**		

CADM1	Cell Signaling Technology	Cat# 30994, RRID:N/A
HRP-conjugated anti-rabbit IgG	Cell Signaling Technology	Cat# 7074, RRID: AB_2099233

**Biological samples**		

Human pancreatic islets from healthy donors and donors with T2D	Nordic Network for Clinical Islet Transplantation, Human Tissue Laboratory, EXODIAB/LUDC (http://www.nordicislets.org).	N/A

**Critical commercial assays**		

AllPrep DNA/RNA kit	Qiagen	Cat#80204
miRNeasy Mini Kit	Qiagen	Cat#217004
High Sensitivity D1000 ScreenTape	Agilent	Cat#5067-5584
High Sensitivity D1000 Reagents	Agilent	Cat#5067-5585
QIAseq miRNA library prep kit	Qiagen	Cat#331502
Human insulin ELISA	Mercodia	Cat#10-1113-10
High Range Rat Insulin ELISA	Mercodia	Cat#10-1145-01
Pierce™ BCA Protein Assay Kit	Thermo Fisher Scientific	Cat#23225
miRNeasy RNA purification kit	Qiagen	Cat#217084
High-Capacity cDNA Reverse Transcription Kit	Thermo Fisher Scientific	Cat#4374966
TaqMan™ Advanced miRNA cDNA Synthesis Kit	Thermo Fisher Scientific	Cat#A28007
TaqMan™ Universal Master Mix II (no UNG)	Thermo Fisher Scientific	Cat#4440043
TaqMan™ Universal PCR Master Mix	Thermo Fisher Scientific	Cat#4304437
4-15% Mini-PROTEAN TGX Stain-Free gels	Bio-Rad	Cat#4568083
Low Fluorescence PVDF Membrane	Bio-Rad	Cat#1620260
Clarity Western ECL Substrate	Bio-Rad	Cat#1705061

**Deposited data**		

Small RNA-sequencing data	Lund University Diabetes Center (LUDC) repository (https://www.ludc.lu.se/resources/repository) with accession number: LUDC microRNA cohort (LUDC2025.08.2)	N/A
scRNA-sequencing data	Gene Expression Omnibus with accession number: GSE153855)	N/A

**Experimental models: Cell lines**		

Human: EndoC-βΗ1 β-cells	21865645	RRID: CVCL_L909
Rat: INS-1 832/13 β-cells	10868964	RRID: CVCL_7226

**Oligonucleotides**		

See Table S12 for Oligonucleotide details	N/A	N/A

**Software and algorithms**		

FastQC (v0.11)	https://www.bioinformatics.babraham.ac.uk/projects/fastqc/	RRID:SCR_014583
UMI-Tools (v1.0.1)	28100584	RRID:SCR_017048
Cutadapt (v3.7)	28715235	RRID:SCR_011841
Trimmomatic (v0.39)	24695404	RRID:SCR_011848
STAR (v2.7.2)	23104886	RRID:SCR_004463
featureCounts from Subread (v1.6.4)	24227677	RRID:SCR_009803
miRge3.0	34308351	N/A
Deseq2 (v1.34)	25516281	RRID:SCR_015687
limma (v3.62)	25605792	RRID:SCR_010943
MASS (v7.3)	https://cran.r-project.org/web/packages/MASS/index.html	RRID:SCR_019125
miRBaseConverter (v1.18)	30598108	RRID:SCR_023873
hypeR (v1.10)	31498385	N/A
WGCNA (v1.70)	19114008	RRID:SCR_003302
WebGestaltR (v0.4.4)	https://cran.r-project.org/web/packages/WebGestaltR/index.html	RRID:SCR_024312
biomaRt (v2.62)	19617889	RRID:SCR_019214
Adobe Illustrator 2020	Adobe (https://www.adobe.com/products/illustrator.html)	RRID:SCR_010279
R (v4)	http://www.r-project.org/	RRID:SCR_001905
GraphPad Prism 9	http://www.graphpad.com/	RRID:SCR_002798

### Experimental model and study participant details

#### Human islets

Human islets of Langerhans from cadaveric donors were procured from the Human Tissue Laboratory, a collaboration between Excellence in Diabetes Research (EXODIAB) in Sweden (www.exodiab.se) and the Nordic Network for Clinical Islet Transplantation (www.nordicislets.org). Informed consent for research purposes of all islet material used in this study was in place for all donors or their next of kin, and all procedures had prior approval by the Swedish Ethical Review Authority (Dnr 2011/263). Pancreata were initially processed by enzymatic digestion and thereafter pancreatic islets were purified by density gradient separation. Islet count and purity were determined as previously described,^58^ and islet preparations with purity ≥80% were used in our experiments. Islets were derived from 38 male (33 ND/5 T2D) and 23 (19 ND/4 T2D) female donors, and sex was accounted for as a covariate during our miRNA differential expression analysis. We demonstrated that sex is associated with miRNA expression, as we identified 5 miRNAs that were related to sex. More details about donor characteristics, including age, are presented in Table S1. Data on ancestry, race, ethnicity, and socioeconomic status were not collected in this study.

#### RNA isolation and library preparation

RNA was extracted from human pancreatic islets using the AllPrep DNA/RNA kit or the miRNeasy Mini Kit (#80204/217004, Qiagen, Germany) and stored at −80°C. RNA concentration was then measured by Nanodrop ND-1000 (Thermo Fisher Scientific, USA), and RNA integrity was determined using the Agilent 2200 TapeStation- High sensitivity D1000 ScreenTape assay (Agilent, Germany). Finally, miRNA-seq cDNA libraries were constructed from 10 ng high-quality RNA using the QIAseq miRNA library prep kit (#331502, Qiagen, Germany) as per the manufacturer’s instructions and sequencing was performed on an Illumina NextSeq 500 platform (Illumina, USA).

#### Global small RNA sequencing processing

We performed small RNA sequencing (sRNA-seq) in human islets from 61 donors, 9 of which derived from donors with type 2 diabetes (T2D) and 52 from non-diabetic (ND) donors (donor information included in Table S1) to define the miRNA profile of human islets. Out of 2884 miRNA sequences in miRBase, 715 were expressed in our human islet samples (Table S10). Following quality control, an in-house analysis pipeline was employed to map and quantify the generated sequence reads (mean sequence depth: ≈29 million reads/sample) to annotated miRNAs using miRBase (v22) as a reference miRNome.^59^ Quality control was performed on the sRNA-seq reads using FastQC (v0.11).^60^ The downstream processing workflow included: i) extraction and storage of miRNA Unique Molecular Identifiers (UMIs) and removal of 3′ adapters, ii) removal of 5′ adapters, iii) retention of reads with proper size and quality, and iv) quantification. The UMIs, which are included in the QIAseq miRNA library kit, are 12bp sequences that allow the identification of PCR duplicates during sample preparation. They reside after the 3′ adapters in the miRNA read, which in turn are located before the miRNA sequence. The 3′ adapters (5′-AACTGTAGGCACCATCAAT-3′) were identified and removed, allowing for two mismatches, and the UMI sequence was stored for each read with UMI-Tools (v1.0.1). Removal of the 5′ adapters (5′-GTTCAGAGTTCTACAGTCCGACGATC-3′) was performed with Cutadapt (v3.7) with a maximum error rate = 0.2. Trimmomatic (v0.39) was used to apply a sliding window filtering of 4 bp to remove bases with average quality below 25, while reads with less than 18bp or more than 30bp were also removed. The reads were then mapped and annotated to the human miRNAome as provided by miRBase (v22) using STAR (v2.7.2). Mapping parameters included end-to-end alignment (no soft-clipping), a minimum number of nucleotides to be aligned = 16, a maximum number of multiple alignments = 10, and allowing for 2 mismatches. Only reads that mapped on the forward strand were retained using featureCounts from Subread (v1.6.4). Reads with identical UMIs were collapsed to one read before they were counted.

Small RNA sequence reads that passed quality control were also processed with miRge3.0^61^ trimming for the 3′ adapter sequence (5′-AACTGTAGGCACCATCAAT-3′) as well as extracting the QIAseq UMIs in order to remove PCR duplicates. We found quantification of miRNA expression with this tool was strongly correlated with the miRNA count pattern observed in our in-house pipeline (regression model r^2^ = 0.97, *p* < 2.2x10^−16^), suggesting that these two methods are highly comparable (Figure S9). Therefore, although our in-house pipeline for miRNA quantification allowed us to apply a more updated miRBase database version for mapping and to adjust and set stricter quality control and mapping thresholds (see *above*), we were confident in exploiting miRge3 to further mine our human islet sRNA-seq dataset, and appropriate options to additionally obtain novel miRNAs and A-to-I editing sites were selected. Detected A-to-I modifications in the sequences of expressed miRNAs correspond to A-to-G changes in the sequenced cDNA fragments. An A-to-I modification is defined as robust and reported if it is present in at least 80% of samples in each of the ND and T2D sample groups.

#### Differential miRNA expression

Differential miRNA expression between categorical variables (T2D vs. ND, Female vs. Male) was determined by Wald significance tests as applied by Deseq2 (1.34).^62^ Before analysis, miRNAs with less than 1 count in more than 50% of the samples were filtered out. The raw miRNA expression data that passed the filtering step were added into a Deseq2 generalized linear model corrected for sequencing batch effects, islet purity, and donor sex (T2D vs*.* ND), or sequencing batch effects and islet purity (Female vs*.* Male).

#### Dynamic glucose-stimulated insulin secretion in human islets

Glucose-stimulated insulin secretion was assessed from human donor islets using a dynamic glucose perifusion system. All experiments were performed by Olle Korsgren and colleagues at Uppsala University, and subsequently the resulting data were provided by the Human Tissue Lab (see acknowledgments). Islets were initially perfused with low glucose (1.76 mM) for a minimum of 30 min. Perifusion was then continued with the same glucose concentration for 4 min and thereafter with high glucose (20 mM) for at least 22 min. For a subset of the measurements (*n* = 27 ND and 5 T2D donors) the perifusion continued for 42 min making it possible to estimate second-phase insulin secretion. Samples were collected for insulin measurement every minute (or every 6 min at time-points>22 min) after the 30 min initialisation period.^63^ First and second-phase insulin secretion were thereafter determined by comparing AUC measurements for ND versus T2D donors (Phase 1: *n* = 49 ND and 8 T2D donors and Phase 2: *n* = 27 ND and 5 T2D donors).

#### Gene/miRNA correlations with continuous variables

For correlations of miRNA/mRNA data with different variables (AUC of first- and second-phase insulin secretion, HbA1c, BMI) two different statistical methods were used. This was to ensure that low statistical power did not prevent the discovery of probable outcomes. Insulin secretion, miRNA expression and mRNA expression data were derived from the same donors, while different batches of islets were used for each parameter. Significant correlations were defined as those with a nominal *p*-value <0.05 by a likelihood-ratio test (LRT) and a linear regression analysis with robust standard errors. Likelihood ratio test (LRT) was performed with Deseq2 (v1.34). Statistical significance (*p*-values) was determined by the difference in deviance between a ‘full’ model, consisting of all factors (sequencing batch, islet purity, sex, variable under study) and a ‘reduced’ model, consisting of the ‘full’ model factors except the variable under study. LRT thus indicates whether the term removed in the ‘reduced’ model can explain a significant amount of variation in the data. As input, raw values for the dependent variable (miRNA/mRNA expression counts) were used. Secondly, analyses were also performed by fitting a linear model by robust regression using an M estimator in the R package, MASS. Input miRNA/mRNA counts were normalised using variance stabilizing transformation before removing the sequencing batch effects, purity, and sex using the R function “*removeBatchEffect”* from the limma R package.^64^ Log-transformation was also applied within the model for all expression and AUC data analyzed.

#### T2D-associated miRNA dataset integration

DE miRNAs between non-diabetic and T2D donors described in previous studies^18^^,^^19^^,^^20^^,^^21^ were collected and compared after updating the miRNA names to those in the most recent miRBase version (v22) using miRBaseConverter (v1.18) in R. As these comparative studies selected more stringent thresholds to define DE miRNAs, we selected DE miRNAs with a minimum of a 2-fold change in expression in either direction for the comparisons. This resulted in a subset of 17 miRNAs (15 upregulated/2 downregulated).

#### T2D-associated gene dataset integration

A comprehensive list of DE genes in T2D was generated using data from a number of previous studies including our own comparing islets from non-diabetic and T2D donors either using a bulk RNA-seq^22^^,^^23^^,^^24^^,^^27^^,^^28^ or a single-cell RNA-seq approach.^25^^,^^26^^,^^29^ Further processing involved two steps. Genes with available Ensembl IDs were included directly in the list, otherwise, gene symbols were matched to their Ensembl IDs using the R package biomaRt (GRCh38). Genes with no assigned Ensembl ID were scanned for aliases with biomaRt and a second round of Ensembl ID matching was performed. Genes without Ensembl IDs after the second matching step were not included in the list, while genes matched to multiple Ensembl IDs were represented only by their first matched Ensembl ID.

#### Enrichment analysis

Enrichment analysis was performed to identify over-represented disease terms associated with a given miRNA set (background: all miRNAs in the corresponding disease database) and over-represented miRNAs that target a specific set of T2D-related genes (background: all genes negatively associated with miRNAs; *n* = 10,535), using the hypergeometric tests in the R package hypeR (v1.10).^65^

#### miRNA-mRNA target identification

We used our already published human islet transcriptomic data^22^ for identification of reliable miRNA mRNA targets in human islets. From our human islet cohort, we extracted a subset in which we had both miRNA and mRNA expression data (*n* = 24). MiRNAs typically exert negative control on gene expression. Hence, we first identified miRNA-mRNA pairs that had significant negatively correlated expression levels after correcting for donor sex and islet purity. To strengthen the robustness of the analysis, only miRNA-mRNA pairs with experimentally validated interactions included in the miRTarBase (v8)^66^ and TarBase (v8)^67^ databases, or with strongly predicted direct interactions based on the well-established algorithms of TargetScan (v8)^68^ and miRDB (v6)^69^ databases, were retained.

#### Weighted correlation network analysis (WGCNA) and pathway enrichment analysis

To identify co-expression networks between the mRNA targets of miRNAs in human islets, a set of high-quality human islet RNA-seq data derived from 219 donors^22^ was processed with the R package WGCNA (v. 1.70).^32^ The analysis was performed using the blockwiseModules function with parameters: power = 20, networkType = “signed”, minModuleSize = 50, mergeCutHeight = 0.3, pamRespectsDendro = F, corType = “bicor”, maxPOutliers = 0.1. Default settings were selected for the rest of the parameters. First, a similarity matrix was constructed by calculating the correlation coefficient of each gene using biweight midcorrelation. As input, gene expression values were transformed using a variance stabilizing transformation. By assigning only positively correlated genes as connected (signed network type and default by WGCNA) the similarity network was transformed into an adjacency matrix using a power function. After examining a range of power values and evaluating the corresponding scale independence and mean connectivity plots, a soft-thresholding power of 20 was selected in order to achieve a scale-free network with a degree of independence above 0.80. Next, a topological overlap matrix was generated from the adjacency matrix and hierarchical clustering took place using a dynamic tree-cut algorithm to identify co-expression gene clusters.

Pathway over-representation analysis was performed in the co-expressed mRNA targets of each cluster with the R package WebGestaltR (v. 0.4.4) using the Gene Ontology Biological Process database. The enriched pathways for each cluster were sorted by pFDR and the top 5 non-overlapping terms with the lowest pFDR were illustrated.

#### Processing of single-cell transcriptomic sequencing

Single-cell RNA sequencing data from 6 human islet ND donors was downloaded from the GEO database (GEO: GSE153855). Gene expression values from 311 β-cells were processed by first updating the gene symbols in R using the biomaRt package and then by defining expressed genes as those expressed in at least one β-cell with mean Reads Per Kilobase Million (RPKM) values higher than 1. Single-cell RNA sequencing data from a further 5 human islet T2D donors was next downloaded from the same dataset (GEO: GSE153855). To identify β-cell enriched miRNAs in ND and T2D islets, differential miRNA expression analysis was performed (β-cell vs. α-cell) in both the ND islet group data and the T2D islet group data with Wald significance testing as applied by Deseq2 (v. 1.34).^62^

#### INS-1 832/13 and EndoC-βh1 cell culture

Rat insulinoma derived INS-1 832/13 β-cells^70^ were cultured in RPMI 1640 media (11.1 mmol/L D-glucose) supplemented with 10 mmol/L HEPES, 2 mmol/L L-glutamine, 1 mmol/L sodium pyruvate (Hyclone, Cytiva, Uppsala, Sweden), 10% fetal bovine serum (FBS) and 50 μmol/L β-mercaptoethanol (Sigma-Aldrich, Stockholm, Sweden). Human fetal derived EndoC-βh1 cells^71^ were cultured in Matrigel/fibronectin coated (100 and 2 μg/mL, respectively, Sigma-Aldrich) vessels in DMEM (Gibco, Thermo Fisher Scientific, Stockholm, Sweden) containing 5.6 mmol/L glucose, 2% BSA fraction V, 10 mmol/L nicotinamide, 50 μmol/L 2-mercaptoethanol, 5.5 μg/mL transferrin, 6.7 ng/mL sodium selenite, 100 U/mL penicillin, and 100 μg/mL streptomycin (Sigma-Aldrich). Both cell lines were maintained in a humidified atmosphere with 5% CO2 at 37°C.

### Method details

#### miR-9, miR-187 and miR-101-3p overexpressed control (CTRL), glucotoxicity (GT) or glucolipotoxicity (GTL) induced β-cell models

INS-1 832/13 cells (200,000 cells/well/24 well plate) were transiently transfected with PremiR miRNA Precursors (pre-miRs) targeting miR-9-5p, miR-187-3p or miR-101-3p (Cat no: AM17100, Assay IDs:PM10022, PM10633 and PM11414 respectively, Thermo Fisher Scientific, Stockholm, Sweden) at 1 nmol/L. In each case, transfections were performed with lipofectamine RNAiMAX transfection reagent (Thermo Fisher Scientific), and a non-targeting pre-miR control (Cat no.: AM17110, Thermo Fisher Scientific) at 1 nM was used as a negative control. Cells were cultured as described in the presence of the selected pre-miRs for 48 h. Thereafter, cell culture media was replaced and transfected cells were either preincubated for a further 24 h with media supplemented with 11.1 mmol/L glucose as previously in culture (CTRL) (miR-9-5p, miR-187-3p and miR-101-3p transfected cells), with 16.7 mmol/L glucose (GT) or with 16.7 mmol/L glucose and 0.5 mmol/L palmitate (GLT) (miR-101-3p transfected cells only) before qPCR and insulin secretion analysis were performed. EndoC-βH1 cells were transiently transfected with PremiR miRNA Precursors (pre-miRs) targeting hsa-miR-101-3p (Cat no: AM17100, Assay ID:PM11414, Thermofisher Scientific) and a Precursor Negative Control #1 (Cat no: AM17110, Thermofisher Scientific), both at 1 nmol/L following the same procedure as for INS-1 832/13 cells.

#### RNA extraction and qPCR

Total RNA was extracted from INS-1 832/13 and EndoC-βH1 cells using miRNeasy RNA purification kit (Qiagen) as per manufacturer instructions, and RNA concentration and purity were assessed by NanoDrop ND-1000 UV-vis spectrophotometer (Thermo Fisher Scientific). Pre-designed TaqMan expression assays (Thermo Fisher Scientific) for miR-9-5p, miR-187-3p and miR-101-3p (Assay IDs: 000583, 001198 and 002253, respectively for INS-1 832/13 cells and Assay ID (hsa-miR-101-3p): 002253 for EndoC-βH1 cells) and for selected target mRNAs (*Cadm1, CADM1, Clasp2, Exoc8, Ifg1r, Ppp1cb, PPp1r12a, Sec62 and Stard13* (Assay IDs: Rn_0000457556_m1, Hs00942509_m1, Rn_01447771_m1, Rn_00594566_s1, Rn_00583837_m1, Rn_00565033_m1, Rn_00588037_m1, Rn_01493449_m1 and Rn_01402162_m1 respectively), were employed for expression analysis. RNA for each sample was reverse-transcribed to cDNA with High-Capacity cDNA Reverse Transcription Kit (Thermo Fisher Scientific) on Applied Biosystems 7900HT real-time reverse transcription-polymerase chain reaction (RT-PCR) system as per manufacturer protocol. Thereafter standard RT-PCR was performed with either TaqMan Universal Master Mix II (no UNG) for miRNA assays or TaqMan Universal PCR Master Mix (Thermo Fisher Scientific) for mRNA assays on a ViiA7 RT-PCR system (Thermo Fisher Scientific), using default cycling parameters.

Samples requiring comparison were run in the same 384 well plate. To serve as controls for normalization, predesigned TaqMan gene expression assays (Thermo Fisher Scientific) for rat endogenous U6 snRNA and U87 snoRNA (Assay ID: 001973 and 001712, respectively) or Hprt1 and Ppia (Rn_01527840_m1 and Rn_00690933_m1, respectively) or for human endogenous RNU44 and RNU48 (Assay ID: 001094 and 001006, respectively) or HPRT1 and PPIA (Hs02800695_m1 and Hs04194521_s1, respectively) as appropriate were assessed in each sample within each experimental run when investigating miRNA or mRNA target expression, respectively. Finally, expression levels were determined by the comparative Ct method (ΔΔCt Method) and expression of each gene of interest was expressed relative to expression of the control genes.

#### Western blot analysis

MiR-101-3p overexpressed INS-1 832/13 cells were washed with ice-cold PBS and lysed with RIPA buffer supplemented with protease and phosphatase inhibitor cocktails (Complete and PhosSTOP; Roche, Mannheim, Germany) on ice with gentle shaking for 30 min. The cell lysate, stored at −20°C, was thawed and centrifuged at 4°C for 10 min at 10 000 × g before use, and the supernatant was used for analysis. After dilution to a defined concentration (10 μg protein/sample) with RIPA buffer, mixing with 4× Laemmli protein sample buffer (Bio-Rad, Hercules, CA, USA) with 10% 2-mercaptoethanol, and heating at 70°C for 10 min, the protein sample was applied to SDS-PAGE using 4–15% Mini-PROTEAN TGX Stain-Free gels (Bio-Rad) and transferred to an Immun-Blot Low Fluorescence PVDF Membrane (Bio-Rad) using a Trans-Blot Turbo Transfer System (Bio-Rad). The gels were activated with UV light for 1 min before blotting to produce tryptophan-dependent fluorescence, and total protein on the membrane after transfer was analyzed before blocking with 3% BSA in TBS-T. Incubation with primary antibody for CADM1 (Cell Signaling Technology, Danvers, MA, USA; Cat# 30994, RRID:N/A, 1:1000) was performed overnight at 4°C. Thereafter, incubation with secondary antibody HRP-conjugated anti-rabbit IgG (Cell Signaling Technology, Cat# 7074, RRID: AB_2099233; 1:2000) was performed for 1 h at room temperature. Clarity Western ECL Substrate (Bio-Rad) was used for visualization of proteins with a ChemiDoc XRS+ System (Bio-Rad). The signal intensity of each protein band was measured using Image Lab software (ver. 6.1; Bio-Rad) and normalized to that of the total protein bands in the lane, which correspond to the total protein volume.

#### Insulin secretion and content analysis

INS-1 832/13 cells were washed twice and then pre-incubated for 2 h in pre-warmed secretion assay buffer (SAB; 114 mmol/L NaCl, 4.7 mmol/L KCl, 1.2 mmol/L KH2PO4, 1.16 mmol/L MgSO4, 25.5 mmol/L NaHCO3, 20 mmol/L HEPES, 2.5 mmol/L CaCl2 and 0.2% BSA, pH 7.3) supplemented with 2.8 mmol/L glucose. Thereafter, the cells were stimulated for 1 h in fresh SAB supplemented with either 2.8 mmol/L glucose or 16.7 mmol/L glucose, and cell supernatants and lysates were collected immediately afterward.

EndoC-βH1 cells were transferred to complete glucose starvation medium containing 2.8 mmol/L for 18 h. Confluent plates were washed twice carefully with 1 mL pre-warmed Secretion Assay Buffer (SAB), pH 7.2 (1.16 mmol/L MgSO_4_, 4.7 mmol/L KCl, 1.2 mmol/L KH_2_ PO_4_, 114 mmol/L NaCl, 2.5 mmol/L CalCl_2_, 25.5 mmol/L NaHCO_3_, 20 mmol/L HEPES and 0.2% Bovine Serum Albumin) containing 1 mmol/L glucose. Cells were then pre-incubated in a new 0.5 mL SAB with 1 mmol/L glucose for 2 h. The cells were stimulated in 0.25 mL SAB at 37°C for 1 h with 1 mmol/L glucose or 20 mmol/L glucose.

To collect cell lysates, cells were washed with PBS and then lysed in RIPA buffer (0.1% SDS, 150 nmol/L NaCl, 1% Triton X-100, 50 mmol/L Tris-HCl, pH 8.0) by shaking on ice at 950 rpm for 30 min before centrifuging at 16 000 g for 20 min at 4°C to remove cell debris. Thereafter, secreted insulin and insulin content in cell supernatants and cell lysates, respectively, were assessed by ELISA (High Range Rat Insulin ELISA, Mercodia, Uppsala, Sweden). For normalization, total protein levels were also assessed in collected cell lysates by BCA (Pierce BCA Protein Assay Kit, Thermo Fisher Scientific, Stockholm, Sweden). All measurements were performed according to the manufacturer’s instructions.

### Quantification and statistical analysis

Data were analyzed and visualized using Prism (GraphPad v9) and R (r-project.org). Data are presented as mean ± SEM. Differentially expressed genes between islets from donors which differ in glucose tolerance condition or sex were defined with Wald significance tests as applied by the R package Deseq2 (1.34). For correlations of miRNA/mRNA data with different variables (AUC of first- and second-phase insulin secretion, HbA1c, BMI), both a likelihood ratio test (performed by Deseq2) and a linear model by robust regression using an M estimator (performed by R package MASS) were used. For comparison of first and second-phase insulin secretion curve measurements, two-way ANOVA with Bonferroni’s post hoc test (insulin curves) and Mann-Whitney tests (AUC graphs) were used. For comparison of insulin secretion/content measurements in the β-cell models two-way ANOVA with Šídák’s multiple comparisons test and paired Student’s t test were used.

## Supplemental information

Document S1. Figures S1–S9

Table S1. Donor characteristics, related to STAR Methods

Table S2. Correlation of miRNA expression with first- and second-phase insulin secretion AUC, related to Figure 1

Table S3. DE miRNAs in T2D, related to Figure 2

Table S4. miRNA expression-disease enrichment analysis for all, upregulated and downregulated miRNAs in T2Drelated to Results (“Enrichment of 34 upregulated T2D-associated miRNAs in miRNA-disease databases”)

Table S5. Associations of miRNAs with donor characteristics (BMI, HbA1c, and sex), related to Figure 2

Table S6. List of mRNA targets of DE miRNAs in T2D, related to Figure 3

Table S7. List of genes previously related to T2D in islet sequencing studies, related to Figure 3

Table S8. WGCNA clusters of mRNA target clusters, related to Figure 4

Table S9. Pathway overrepresentation analysis of mRNA targets within WGCNA clusters, related to Figure 4

Table S10. Associations of miR-101 and miR-9 mRNA targets with first- and second-phase insulin secretion AUCs, related to Figure 6

Table S11. Top abundant miRNAs in pancreatic islets, related to Figure S6

Table S12. Oligonucleotide details, related to key resource file
