# Supplementary material for: Network-based insights into miRNA regulation of β-cell insulin secretion in type 2 diabetes
Source: iScience. 2025 Nov 22;28(12):114200. doi: 10.1016/j.isci.2025.114200 (PMC12721147; doi:10.1016/j.isci.2025.114200)
Supplement: Document S1. Figures S1–S9 [file mmc1.pdf]

iScience, Volume *28*

Supplemental information

Network-based insights into miRNA regulation of β-cell insulin secretion in type 2 diabetes

Elaine Cowan, Alexandros Karagiannopoulos, Alessio Pollastri, Akira Asai, Mototsugu Nagao, Marlena Maziarz, Jonathan L.S. Esguerra, and Lena Eliasson

Figure S1

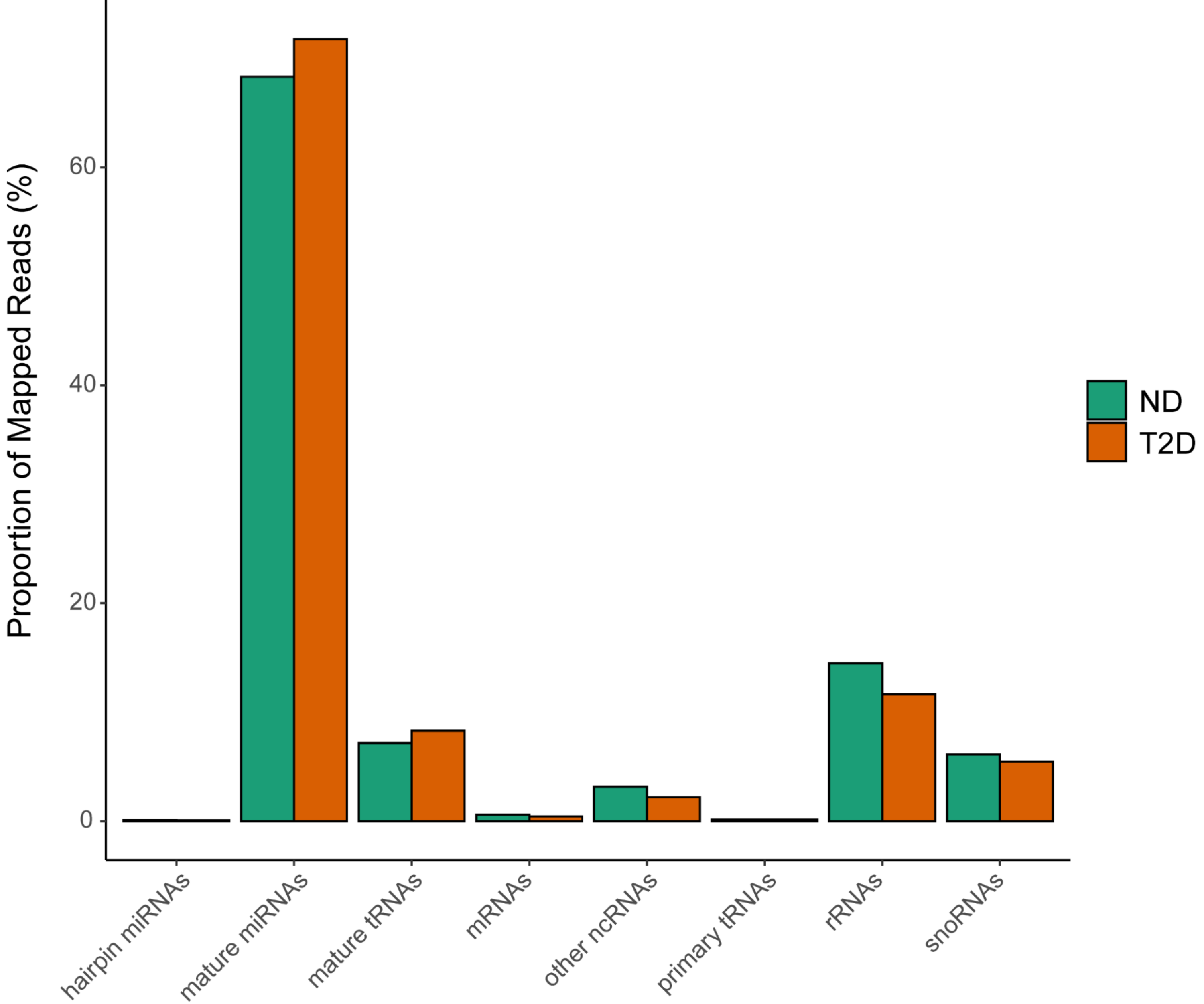

Figure S1. Distribution of RNA types in small RNA sequencing reads. Bar charts representing the percentage of sequence reads mapped to the different RNA types in samples from non-diabetic (ND) and donors with T2D (T2D).

Figure S2

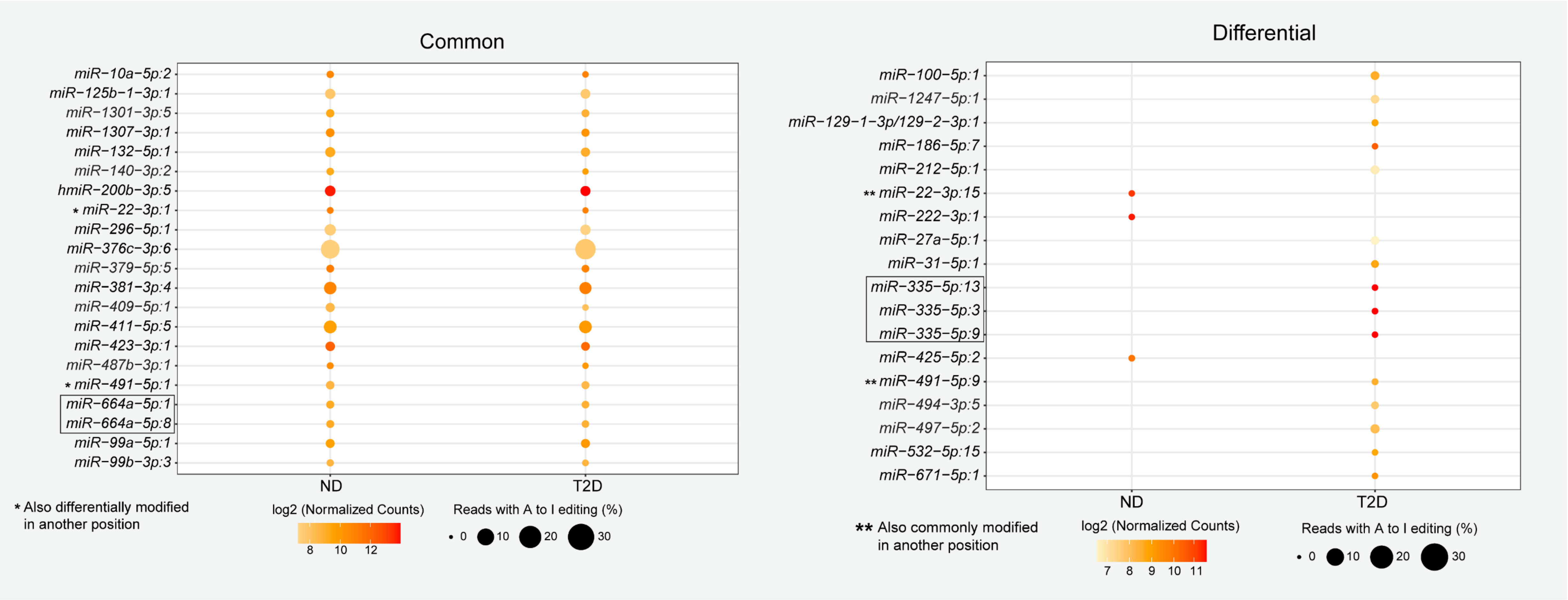

Figure S2. A-to-I modifications in miRNA sequences. Dot plot showing miRNAs with A-to-I modifications in their sequence which are common (left panel) or distinct (right panel) in islets from ND and T2D donors. A given modification is illustrated if is present in >1% sequencing reads and in at least 80% samples. The size of the dots represents the proportion of miRNA sequence reads with an A-to-I modification and the colour scale illustrates the level of miRNA expression in the islets. A single star (*) indicates that a miRNA with an A-to-I modification in both ND and T2D sets is characterized by an A-to-I modification in another position which is prevalent in only one of the two sets. The opposite applies to the miRNAs with two stars (**).

Figure S3

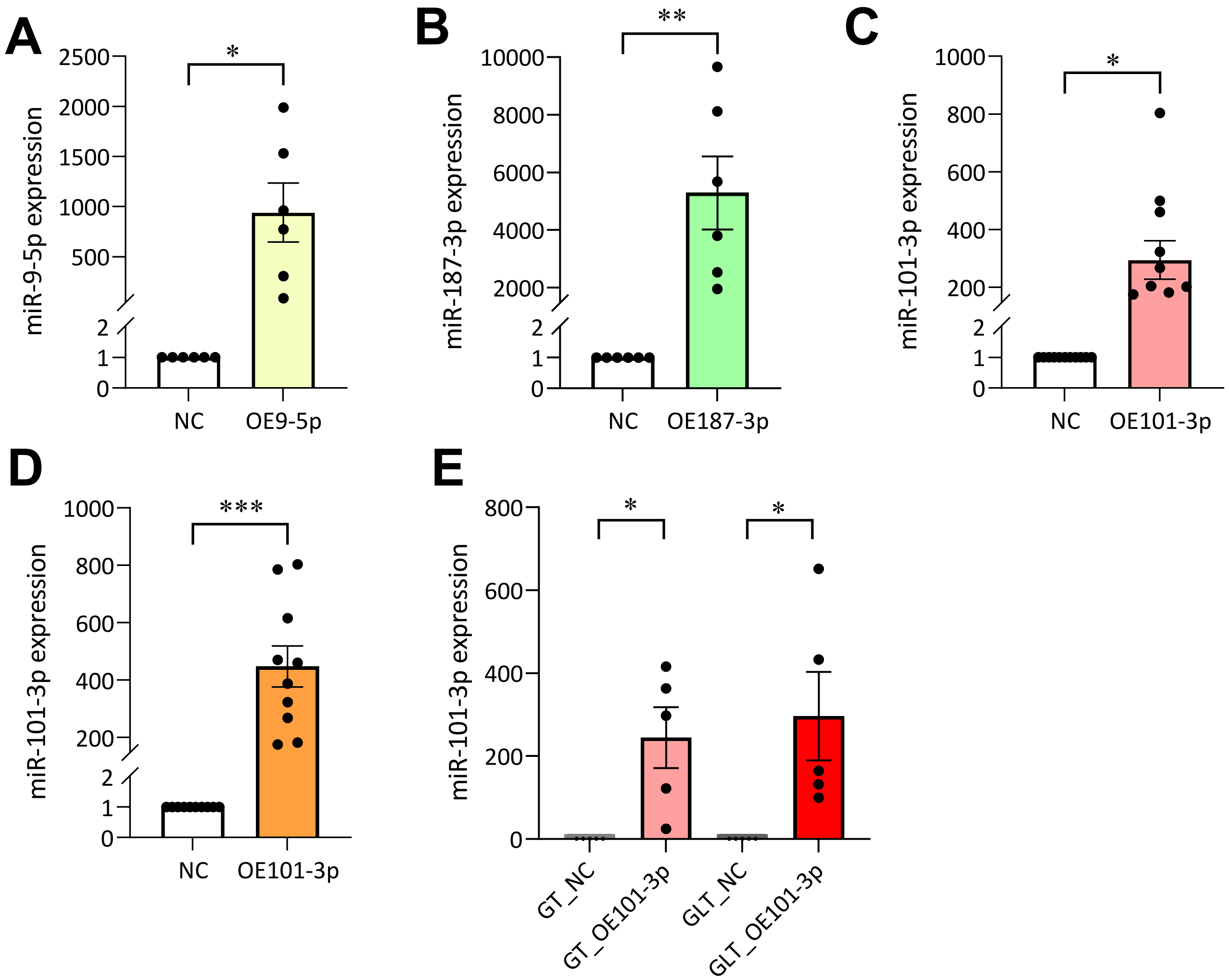

Figure S3. MiRNA overexpression efficiency in β-cell line models. (A) Expression of miR-187-3p in NC and OE9-5p INS-1 832/13 cells. (B) As in A, but OE9-5p. (C) As in A, but OE101-3p. (D) Expression of miR-101-3p in NC and OE101-3p in EndoC-βH1 cells. (E) Expression of miR-101-3p in NC and OE101-3p cells in GT and GLT. NC – negative control; OE9-5p – overexpression of miR-9-5p; OE187-3p – overexpression of miR-187-3p: OE101-3p – overexpression of miR-101-3p; Data are presented as mean±SEM. Statistical analyses were performed using paired Students t-test. *p<0.05; **p<0.01. GT – glucotoxic condition (16.7 mmol/L glucose 24h); GLT – glucolipotoxic condition (16.7 mmol/L glucose and 0.5 mmol/L palmitate 24h).

Figure S4

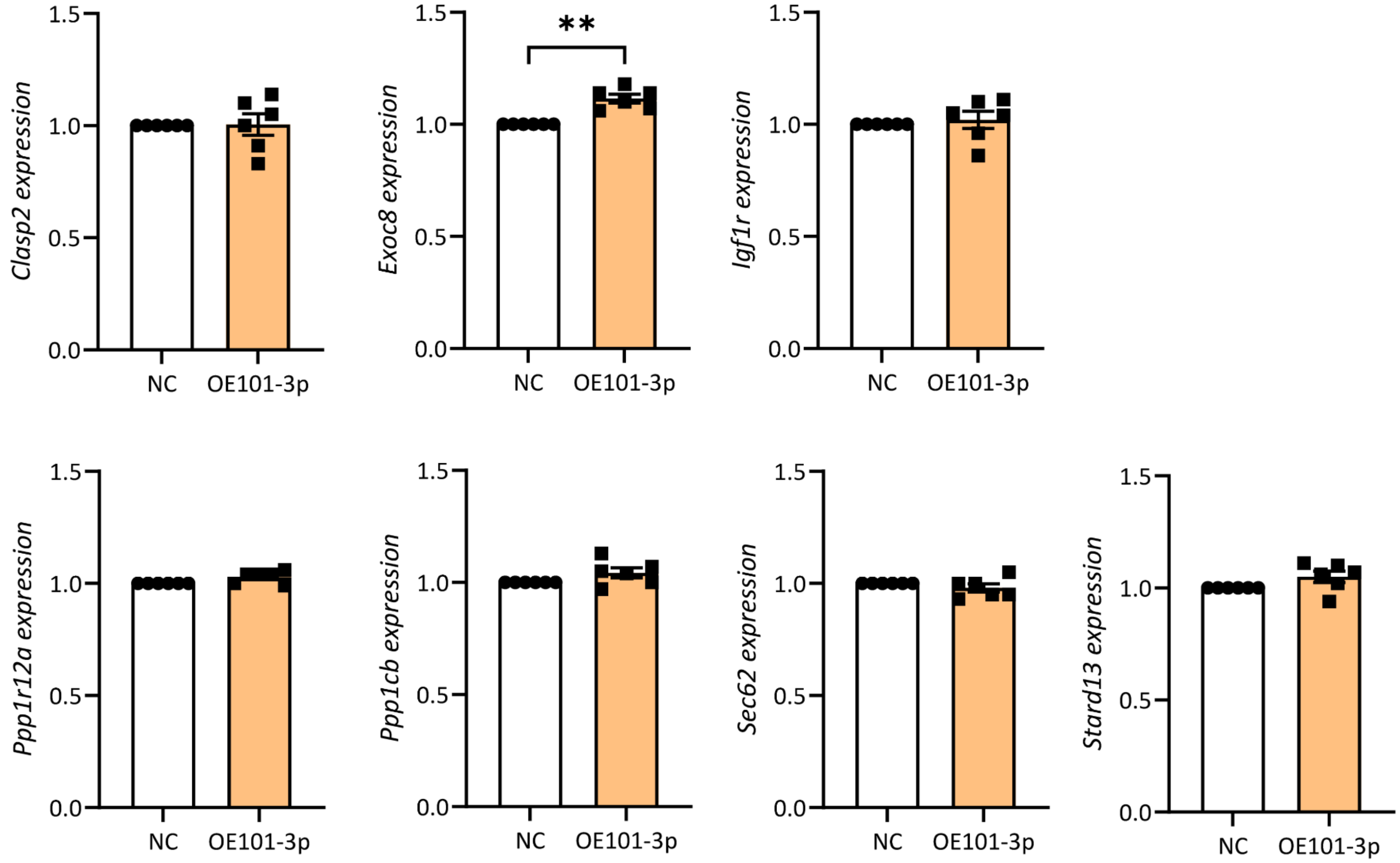

Figure S4. Expression of potential targets of miR-101-3p in INS1-832/13 cells. Gene expression of *Clasp 2, Exoc8, Igf1r,Ppp1r12a, Ppp1cb, Sec62 and Stard13* was measured in NC and OE101-3p INS1-832/13 cells. NC – negative control; OE101-3p – overexpression of miR-101-3p; Data are presented as mean±SEM; $^{**}p<0.01$ using paired Students t-test.

Figure S5

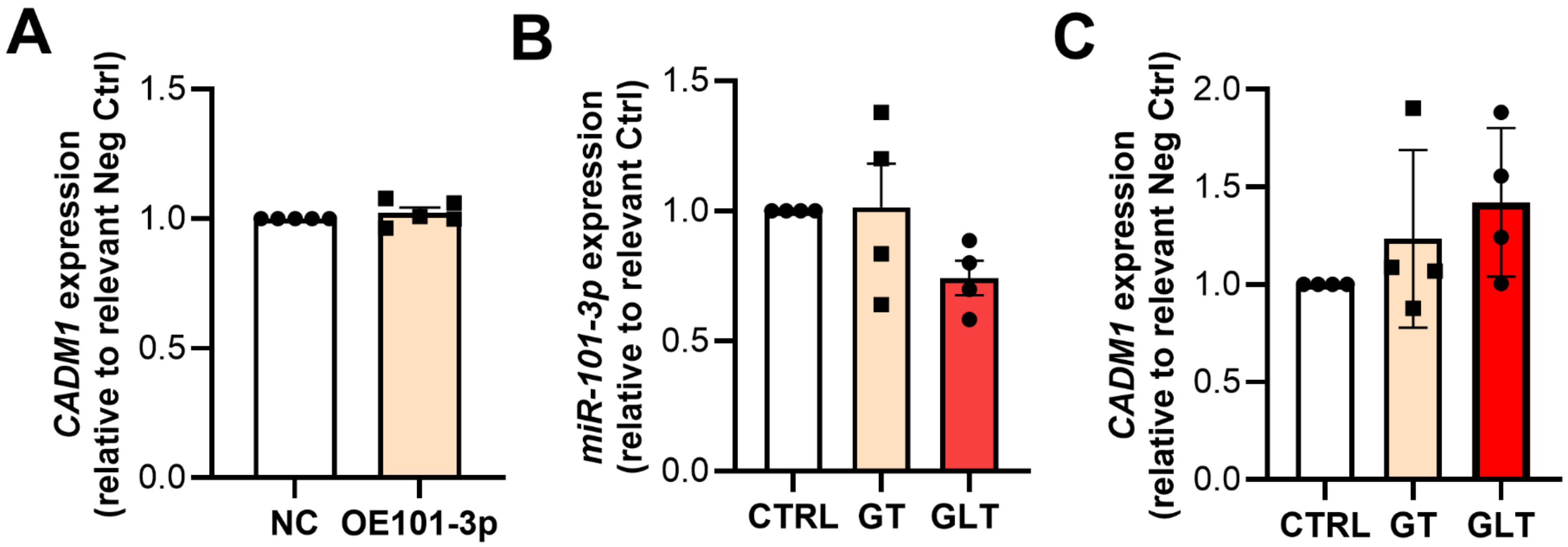

Figure S5. Expression of miR-101-3p and *CADM1* in EndoC-βH1 cells. A. Gene **e**xpression of *CADM1* in NC and OE101-3p EndoC-βH1 cells. B. (**B**) miR-101-3p and (**C**) *CADM1* expression expression in GT and GLT EndoC-βH1 cells. NC – negative control; OE101-3p – overexpression of miR-101-3p; CTRL- control 5.5 mM glucose; GT – glucotoxic condition (16.7 mmol/L glucose 24h); GLT – glucolipotoxic condition (16.7 mmol/L glucose and 0.5 mmol/L palmitate 24h). Data are presented as mean±SEM.

Figure S6

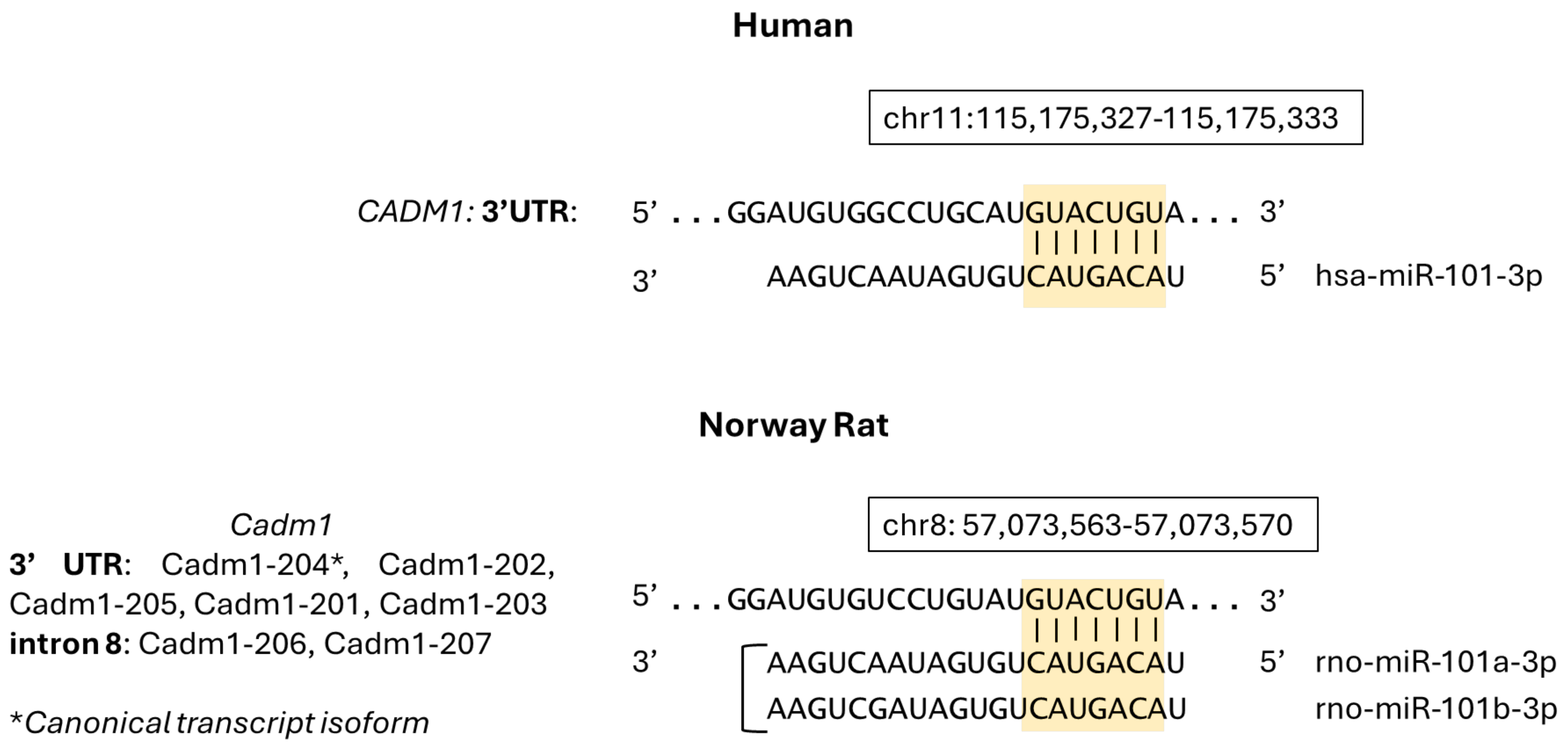

Figure S6. miR-101-3p targets *CADM1* at a conserved genomic site in human and rat. The predicted binding sites between the seed sequence of hsa-miR-101-3p and the human *CADM1* RNA sequence, and between the seed sequences of rno-miR-101a/b-3p and the rat *Cadm1* RNA sequence are shown. In humans, the binding site is located within the 3′ untranslated region (3′UTR) of all CADM1 transcript isoforms, whereas in rats it resides in the 3′UTR of 4 out of 6 Cadm1 isoforms, including Cadm1-204, which is designated as the Ensembl canonical transcript isoform by the Ensembl database (GRCr8 assembly). This flag indicates that Cadm1-204 represents the isoform with the strongest supporting evidence, typically characterized by the highest sequence conservation and expression level. GRCh38 and GRCr8 assemblies were used to define the genomic coordinates for human and rat, respectively.

Figure S7

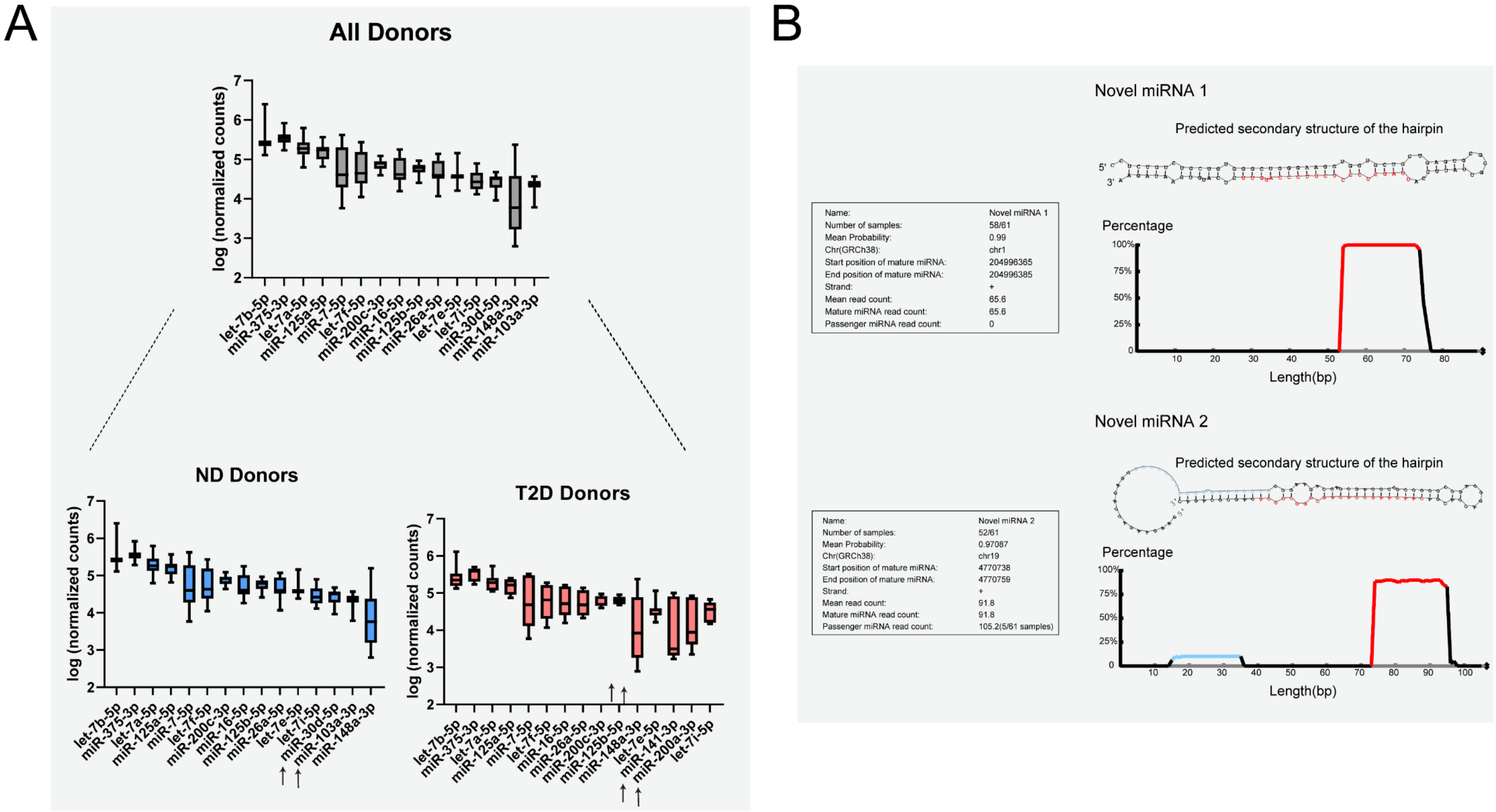

Figure S7. Other miRNA characteristics. (A) Boxplots representing the expression level of the 15 most abundant miRNAs in human islets in all samples and separately for non-diabetic (ND) donors and donors with type-2 diabetes (T2D). Arrows highlight the most abundant miRNAs exclusive to each of the two groups. (B) Two putative islet miRNA sequences were discovered by miRge3 in the majority of our human islet samples. More information on the predicted miRNAs, their predicted secondary hairpin structures, and the percentage of sequence reads mapped to the guide (Novel miRNA 1, Novel miRNA 2) and the passenger miRNA strand (only for Novel miRNA 2) are presented.

Figure S8

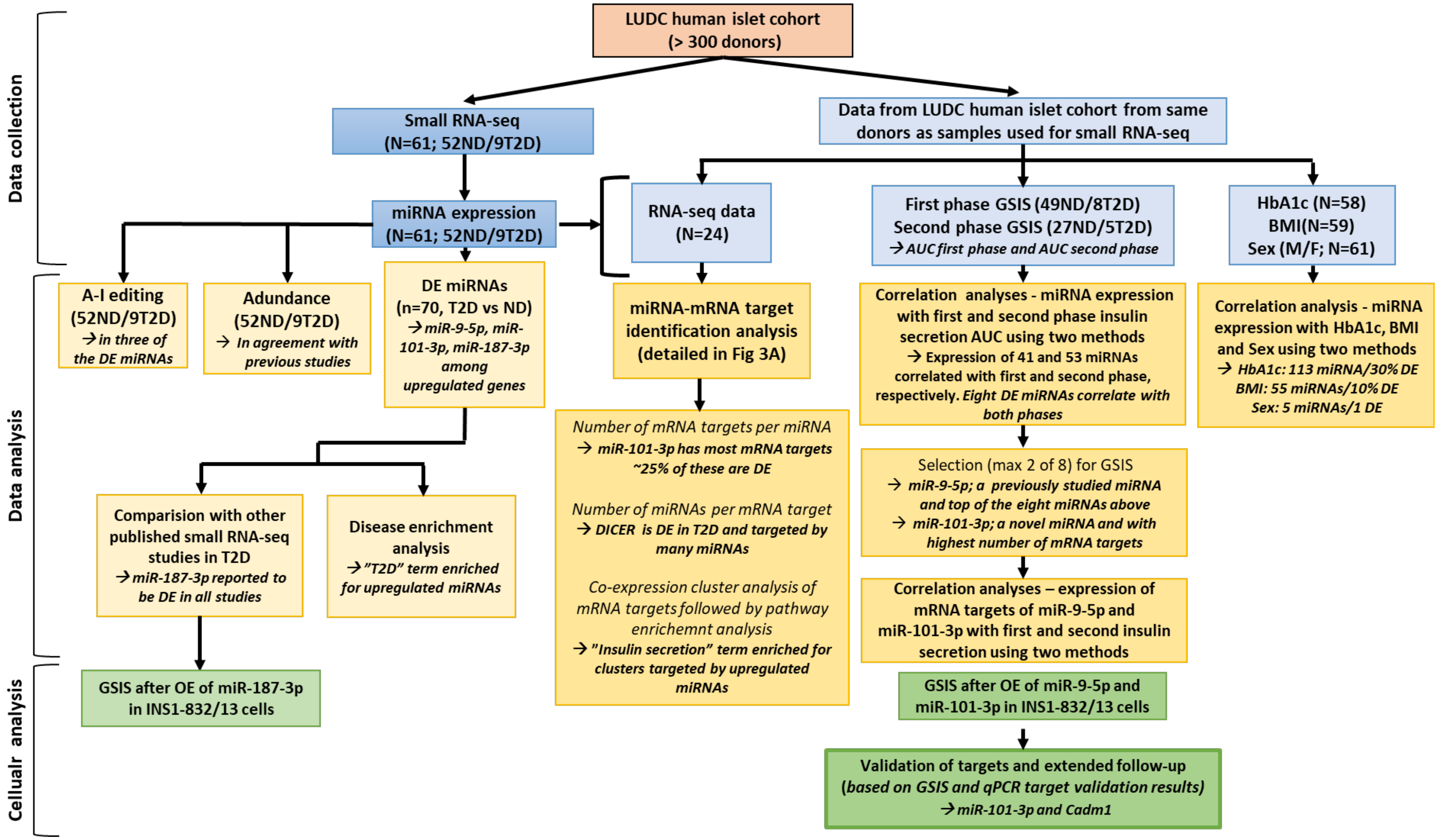

Figure S8. Schematic overview of the study. The workflow illustrates data collection (blue), data analysis (yellow) and cellular analysis (green) procedures, together with a summary of key results, some of which were critical for selecting miRNAs for functional follow-up experiments. For details we refer to the Results and STAR★Methods sections. ND, non-diabetic; T2D, type-2 diabetes; DE, differentially expressed; GSIS, glucose-stimulated insulin secretion; OE,overexpression.

Figure S9

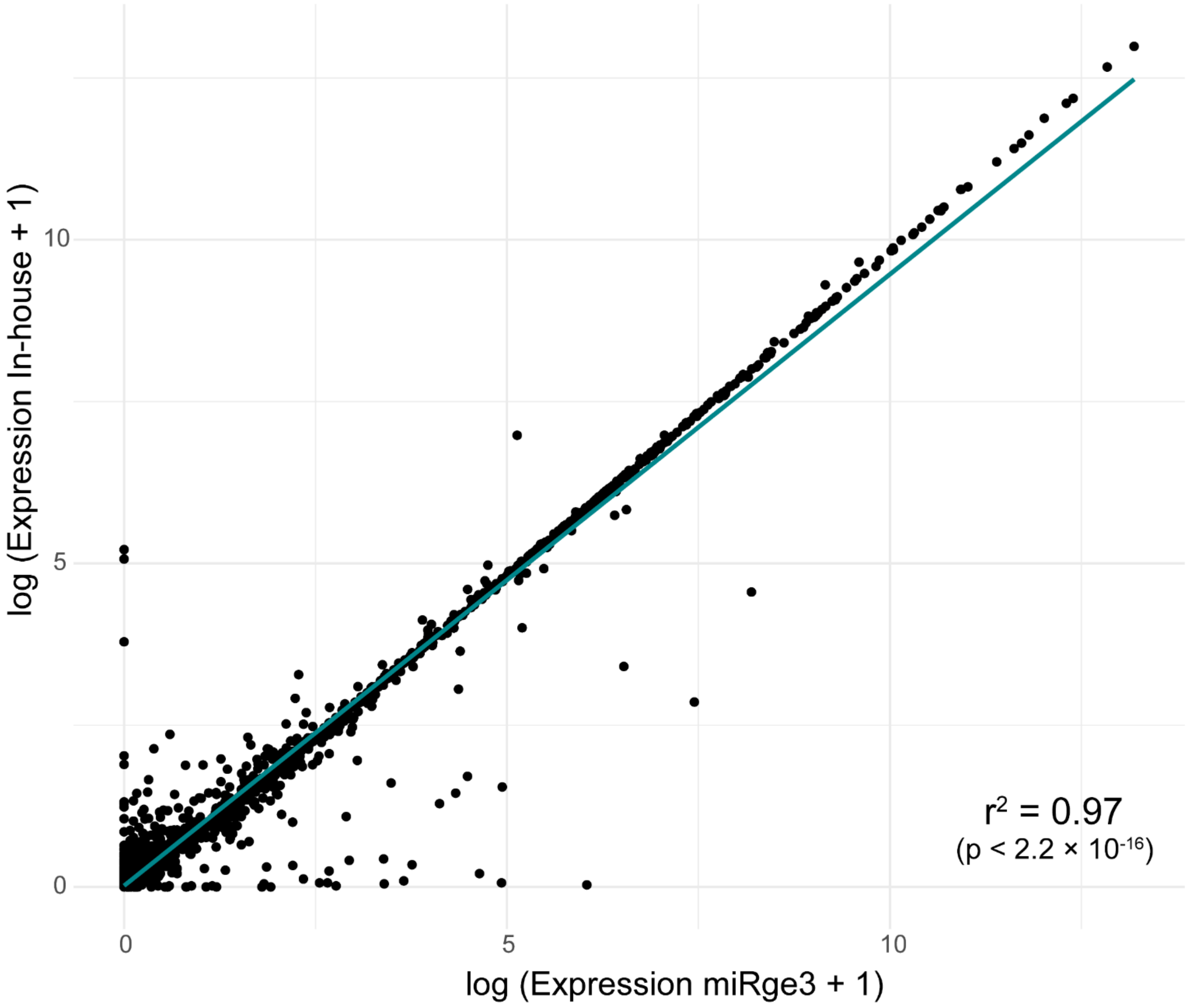

Figure S9. Relationship between islet miRNA expression generated with an in-house pipeline and miRge3. A linear regression model between expression levels of miRNAs produced by the two different methods demonstrates a very high similarity. Log-transformed miRNA normalized counts were used as input.
